# Structure-preserving super-resolution of retinal fundus images via a dual-transformer residual network

**DOI:** 10.3389/fmed.2025.1730678

**Published:** 2026-01-30

**Authors:** Emmanuel Eric Pazo, Salissou Moutari, Fei Gao, Liying Hu, Muhammad Usama, Xiaorong Li, Juping Liu

**Affiliations:** 1 Tianjin Key Laboratory of Retinal Functions and Diseases, Tianjin Branch of National Clinical Research Center for Ocular Disease, Eye Institute and School of Optometry, Tianjin Medical University Eye Hospital, Tianjin, China; 2 School of Mathematics and Physics, Queen's University Belfast, Belfast, United Kingdom; 3 School of Electrical Engineering, Korea Advanced Institute of Science and Technology (KAIST), Daejeon, Republic of Korea

**Keywords:** deep learning, fractal dimension, medical image super-resolution, retinal fundus images, structure preservation, transformer networks, vascular analysis

## Abstract

High-resolution retinal fundus images are critical for diagnosing diabetic retinopathy, yet clinical datasets often contain low-resolution images that obscure fine vascular structures essential for accurate diagnosis. Existing super-resolution methods face a fundamental trade-off: convolutional neural networks produce overly smooth results, while generative adversarial networks (GANs) risk creating hallucinated artifacts. We propose the Dual-Transformer Residual Super-Resolution Network (DTRSRN), a hybrid architecture combining Swin Transformers for global context modeling with a parallel residual Convolutional Neural Network (CNN) pathway for fine-grained vascular detail preservation. Our key innovation lies in using Fractal Dimension analysis to quantitatively measure retinal vascular morphology preservation. Experimental results on three benchmark datasets demonstrate that DTRSRN achieves 33.64 dB PSNR for 2 × super-resolution, out performing state-of-the-art methods including SwinIR (+0.96 dB), HAT (+0.37 dB), and ResShift (+0.30 dB). Critically, DTRSRN achieves 17.0% improvement in vascular structure preservation (Δ*D*_*f*_ = 0.0987) compared to the best baseline, demonstrating superior clinical relevance for retinal image enhancement.

## 1 Introduction

Single Image Super-Resolution (SISR) seeks to reconstruct a high-resolution image I_HR_ from a low-resolution input I_LR_, an ill-posed inverse problem where infinite high-resolution solutions exist. In medical imaging, SISR is a clinical necessity: high-resolution images enable detection of subtle anatomical features such as microaneurysms in retinal images and precise delineation of pathological boundaries. However, acquiring such images is often complicated by hardware limitations, imaging constraints, and the need to minimize patient exposure.

Super-resolution architectures face a fundamental tension between modeling local details and capturing global relationships. Convolutional Neural Networks (CNNs) excel at local features through fixed-size kernels, with foundational works like SRCNN (1) and VDSR (2) advancing to EDSR (3) and RCAN (4). EDSR optimized residual networks by removing batch normalization (BN), while RCAN introduced Residual-in-Residual (RIR) structures with skip connections to focus on high-frequency information.

The restricted receptive field of CNNs motivated Vision Transformers (ViTs) (5), which capture global relationships through self-attention mechanisms (6). SwinIR (7) demonstrated strong performance by combining CNNs for initial feature extraction with Residual Swin Transformer Blocks. This architectural shift revealed a key insight: CNNs excel at local details but fail at global context, while Transformers capture global relationships but may not preserve precise edge features effectively.

Beyond architecture, medical image super-resolution faces an evaluation crisis. Traditional metrics such as PSNR and SSIM (8) are fundamentally inadequate for medical applications (9, 10), as they are insensitive to localized anatomical details and correlate poorly with clinical validity. Models achieving high PSNR scores may still produce clinically significant errors in tumor bound aries or vascular structures. In retinal imaging, fractal dimension (11, 12), a measure of vascular complexity highly sensitive to image quality, reveals limitations that standard metrics fail to capture, highlighting the risk of deploying flawed tools in clinical settings.

This paper introduces the Dual-Transformer Residual Super-Resolution Network (DTRSRN), a hybrid architecture designed to address both the local vs. global feature dilemma and the evaluation crisis. DTRSRN synergistically combines CNNs for detail preservation with Transformers for global context modeling, specifically tailored for retinal fundus imaging. The network optimizes for clinically relevant vascular boundaries and fine textures, employing fractal dimension analysis as a structure-preserving metric to ensure enhanced images maintain the morphological complexity of true high-resolution references.

The primary contributions of this work are summarized as follows:

1) A new hybrid network architecture (DTRSRN) that effectively fuses the detail-capturing capabilities of CNNs with the long-range dependency modeling of Transformers to simultaneously address local and non-local priors in retinal images.
2) A specialized focus on retinal fundus image super resolution, developing a model that overcomes the limitations of general-purpose SR methods by prioritizing clinically relevant vascular features.
3) The introduction of fractal dimension analysis as a clinically relevant evaluation metric quantifies the preservation of retinal vascular complexity, moving beyond traditional PSNR and SSIM metrics.
4) Comprehensive experimental validation demonstrating superior performance in reconstructing clinically significant details, particularly vascular structures essential for diabetic retinopathy diagnosis.

## 2 Related work

### 2.1 Convolutional neural network-based architectures

The application of deep learning to single-image super-resolution (SISR) began with Convolutional Neural Networks (CNNs). Early models such as SRCNN (1) and VDSR (2) were foundational, demonstrating that deep learning could achieve superior performance over traditional methods by learning a non-linear mapping from low-resolution to high-resolution images. The field advanced significantly with architectural optimizations that enhanced network performance and efficiency. EDSR (3) and its multi-scale variant, MDSR, demonstrated that removing unnecessary modules like batch normalization (BN) from the residual network architecture can stabilize training and improve performance. The authors of EDSR also made a key observation about model size, noting that increasing the number of feature channels (width) was often more effective than stacking more layers (depth) for maximizing model capacity under computational constraints.

The next major advancement in CNN-based SR came with the introduction of attention mechanisms. RCAN (4), or Residual Channel Attention Network, introduced a very deep network using a Residual-in-Residual (RIR) structure. This architecture, composed of multiple residual groups with long skip connections and residual blocks with short skip connections, was specifically designed to ease the training of very deep networks. It allows abundant low-frequency information to bypass the main network, thereby forcing the network to focus on learning high-frequency details. The effectiveness of this design was demonstrated in models like Deep Residual Dense Network, which also employed a residual-in-residual dense block (RRDB) strategy. RCAN was optimized using an L1 loss function, a common choice for pixel-level fidelity.

### 2.2 Generative adversarial network-based methods

While PSNR-oriented CNNs excelled at pixel-level accuracy, they often produced images that lacked the realistic textures and fine details of natural images, resulting in perceptually blurry results. This led to the emergence of Generative Adversarial Networks (GANs) for SR, a paradigm shift that prioritized perceptual quality over simple fidelity metrics. SRGAN (13) was a seminal work in this area, building a residual block model and optimizing it within a GAN framework using a perceptual loss.

ESRGAN (14), a significant enhancement of SRGAN, addressed the issue of unpleasant artifacts and other limitations. It introduced three key improvements: first, the network architecture was enhanced by replacing the basic residual blocks with the Residual-in-Residual Dense Block (RRDB), a deeper and more complex structure that removes all batch normalization layers to prevent artifacts and improve generalization. Second, the adversarial loss was improved by adopting a relativistic GAN (RaGAN), which teaches the discriminator to predict “relative realness” rather than an absolute value. This change empowers the generator to recover more realistic textures. Third, the perceptual loss was adjusted to use features from the VGG network before activation, which was empirically found to provide sharper edges and more visually pleasing results than the original approach. ESRGAN's success in the PIRM2018-SR Challenge, where it won first place for perceptual quality, solidified its status as a major milestone.

### 2.3 The emergence of vision transformers in image restoration

The limitations of CNNs' local receptive fields spurred the adoption of Vision Transformers (ViTs) (15) for image restoration. Transformers, originally developed for natural language processing (16) , offered the unique ability to model long-range dependencies across an entire image through self-attention. SwinIR (7) is a strong baseline model in this category. Its architecture consists of a shallow CNN for initial feature extraction, followed by a deep feature extraction module made up of Residual Swin Transformer Blocks (RSTB). SwinIR's efficacy stems from its shifted window mechanism (17), which allows for efficient long-range modeling and content-based interactions between image content and attention weights. This architecture demonstrates a significant advantage over CNNs by achieving superior performance with a fraction of the parameters, sometimes reducing the total number of parameters by up to 67% while still outperforming state-of-the-art CNNs on key benchmarks.

Recent advances in transformer-based super-resolution have pushed performance boundaries further. HAT (18) introduces a hybrid attention mechanism that combines channel attention and window-based self-attention with an overlapping cross-attention module to enhance interaction between neighboring window features, achieving state-of-the-art results on natural image benchmarks. SRFormer (19) proposes permuted self-attention to better balance channel and spatial information, achieving competitive performance with fewer parameters and computations than SwinIR. These methods demonstrate that refinements to the attention mechanism can yield substantial improvements, though questions remain about their effectiveness on specialized domains like medical imaging where local detail preservation is paramount.

Diffusion models have recently emerged as a powerful alternative paradigm for super-resolution. ResShift (20) constructs an efficient diffusion model that transfers between high-resolution and low-resolution images by shifting their residual, substantially improving transition efficiency. With only 15 sampling steps, ResShift achieves performance comparable to or exceeding hundreds-of-steps diffusion methods, demonstrating that careful design of the diffusion process can overcome the traditional speed limitations of generative approaches.

### 2.4 Lightweight CNN architectures and attention mechanisms

Before the rise of transformers, researchers explored lightweight CNN architectures with sophisticated attention mechanisms to improve super-resolution efficiency. PAN (21) introduced pixel attention, a 3D attention scheme that produces attention maps for individual pixels rather than channel-wise or spatial attention vectors. This fine-grained attention mechanism enables PAN to achieve competitive performance with only 272K parameters (17% of SRResNet), demonstrating that efficient attention design can substantially reduce model complexity while maintaining reconstruction quality. Such lightweight approaches remain relevant for resource-constrained deployments, though they lack the global modeling capabilities that transformers provide.

### 2.5 Hybrid CNN-transformer architectures

Recognizing that both CNNs and Transformers have complementary strengths, CNNs for their strong inductive bias on local details and Transformers for their superior global context modeling, the field has recently converged on hybrid models. These architectures are designed to combine the best of both worlds.

One notable example is the Hybrid Network of CNN and Transformer (HNCT) (22) designed for lightweight image super-resolution. HNCT uses a series of Hybrid Blocks of CNN and Transformer (HBCTs) to simultaneously extract both local and non-local priors, demonstrating superior performance and efficiency, a feat recognized in the NTIRE 2022 Efficient SR Challenge. The hybrid attention network for underwater image restoration further illustrates this fusion, employing CNNs for local feature extraction and a Transformer for global relationships, all within a sophisticated multi-attention framework to address complex degradation factors.

Within the specific context of medical imaging, several similar hybrid models have been proposed. One approach for retinal image super-resolution (23) uses a Vision Transformer encoder and a CNN decoder, explicitly acknowledging the high computational complexity of standalone ViTs. The model also employs a progressive training technique and a unique adaptive patch embedding layer. Another compelling work is the Multi-Scale Convolution-Aided Transformer-Based Super Resolution (MSCT-SR) network (24), which uses a dual branch architecture. This network employs one branch for CNNs to capture local details and edge information and another for Transformers to extract global features, explicitly addressing the Transformer's weakness in learning precise edge features. It fuses these representations with a Pyramid Multi-Scale Feature Fusion (PMS-FN) module, capable of effectively reconstructing high-level semantic information and low-level details.

More recently, Zhu et al. (25) proposed a residual dense vision transformer specifically for medical image super-resolution. This approach combines residual dense connections with vision transformers and introduces a segmentation-based perceptual loss that incorporates prior knowledge from medical image segmentation to improve reconstruction of clinically relevant structures. Their work demonstrates the importance of domain-specific design considerations, though performance gains over general-purpose methods suggest that architectural innovations must be accompanied by careful loss function design.

Other related works, such as STAN and skin lesion analysis models, also incorporate hybrid architectures. Interestingly, the literature presents a subtle contradiction: while MSCT-SR uses a CNN branch to address the Transformer's weakness in handling edges, models like STAN claim their use of a Transformer network actually helps improve the extraction of edge information. This demonstrates that the optimal integration of these architectures for preserving clinically critical details remains a subject of active research.

### 2.6 Our method in context

Table 1 summarizes the key differences between DTRSRN and representative baseline methods across architecture, loss functions, and training datasets. Our method uniquely combines a dual-path CNN-Transformer architecture with a clinically motivated fractal dimension loss, specifically targeting retinal fundus images where vascular structure preservation is critical.

**Table 1:** Comparison of super-resolution methods: architecture, loss functions, and datasets.

**Method**	**Architecture**	**Loss function**	**Training data**	**Clinical focus**
SRCNN	CNN (3 layers)	MSE	Natural images	No
EDSR	Deep residual CNN	L1	DIV2K	No
RCAN	Channel attention CNN	L1	DIV2K	No
PAN	Pixel attention CNN	L1	DIV2K	No
SwinIR	Swin Transformer	L1 + Perceptual	DIV2K + Flickr2K	No
HAT	Hybrid attention Transformer	L1 + Perceptual	DIV2K + Flickr2K	No
SRFormer	Permuted attention Transformer	L1	DIV2K + Flickr2K	No
ResShift	Diffusion model	L2 + LPIPS	ImageNet	No
HNCT	Sequential CNN-Transformer	L1	DIV2K	No
MSCT-SR	Dual-branch CNN-Transformer	L1 + Perceptual	Medical images	Partial
RD-ViT	Residual dense ViT	L1 + Seg. perceptual	Medical images	Partial
DTRSRN (Ours)	Parallel dual-path	MSE + Perceptual	Retinal fundus	Yes
	CNN-Transformer	+ Fractal loss	(DRIVE, STARE, HRF)	

Our proposed method is positioned as the next logical step in this architectural progression. While other hybrid models exist, they often focus on general-purpose applications or provide single-faceted solutions to the multi-layered problem of medical image super-resolution. Our work uniquely and comprehensively addresses the specific, high-stakes challenges of this domain through deliberate, synergistic design. It directly confronts the architectural tension between local and global feature extraction while simultaneously addressing the profound limitations of standard evaluation metrics, a concern often overlooked in the development of general-purpose SR models. By doing so, our method seeks to advance the field by creating a model that is not only architecturally sound but also clinically validated and relevant.

## 3 Materials and methods

This section presents the proposed Dual-Transformer Residual Super-Resolution Network (DTRSRN), a hybrid architecture designed specifically for retinal fundus image super-resolution. We detail the network architecture, training strategy, and our fractal dimension-based evaluation framework that ensures clinical relevance.

### 3.1 Problem formulation

The single image super-resolution problem can be formulated as learning a mapping function *F*:*I*_*LR*_→*I*_*HR*_ that reconstructs a high-resolution image $I_{HR}\;\in \;R^{H\times W\times C}\;$from its low-resolution counterpart $I_{LR}\;\in \;R^{h\times w\times c}$, where H = s·h and W = s·w with s being the up sampling factor. For retinal fundus images, this mapping must preserve critical vascular structures while reconstructing fine anatomical details essential for clinical diagnosis.

The degradation model assumes that the low-resolution image is obtained through:


$$\begin{array}{l} I_{\text{LR}}=D\left(I_{\text{HR}}\right)+\eta \end{array}$$

where D(·) represents the degradation operator (typically blur followed by down sampling) and η denotes additive noise. Our objective is to learn the inverse mapping F ≈ D^−1^ while ensuring that the reconstructed vascular structures maintain their morphological complexity.

### 3.2 Dual-transformer residual super-resolution network architecture

**Figure 1:**
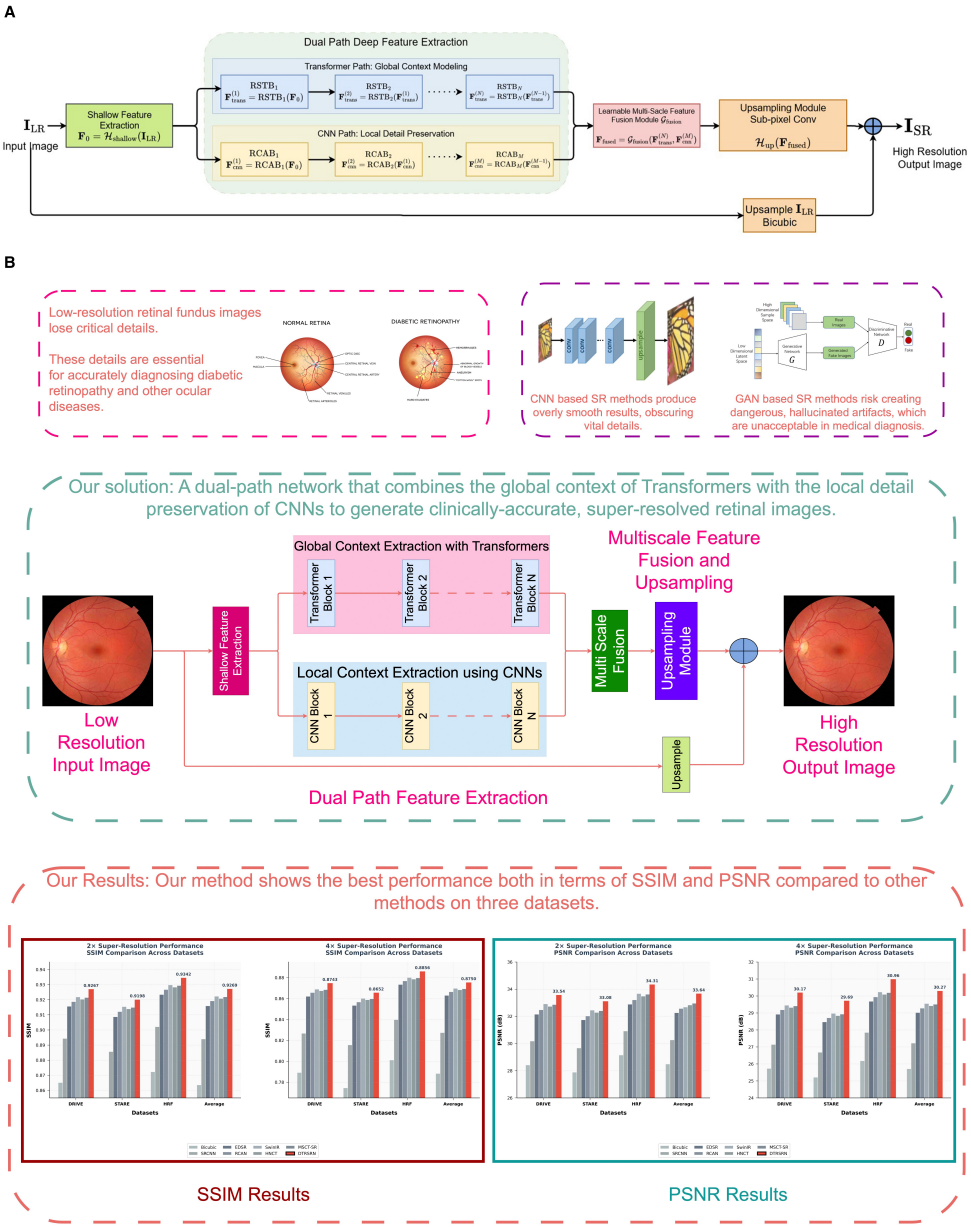
Architecture of the proposed Dual-Transformer Residual Super-Resolution Network (DTRSRN). The network processes input I_L_ through shallow feature extraction H_shallow_ to generate F_0_, which feeds into dual parallel pathways: the Transformer path with N Residual Swin Transformer Blocks (RSTBs) for global context modeling, and the CNN path with M Residual Channel Attention Blocks (RCABs) for local detail preservation. Features ${\text{F}}_{\text{trans}}^{\left(\text{N}\right)}$ and ${\text{F}}_{\text{cnn}}^{\left(\text{M}\right)}$ are fused via learnable multi-scale fusion G_fusion_, then upsampled through sub-pixel convolution H_up_ with residual connection to produce I_S_.

The DTRSRN architecture consists of three main components: a shallow feature extraction module, a dual-path deep feature extraction module that combines Swin Transformers with residual CNNs, and a reconstruction module. Figures 1a and b illustrates the overall network design.

1. Shallow feature extraction: the shallow feature extraction module processes the input low-resolution image to extract initial features while preserving spatial information:
$$\begin{array}{l} F_{0}=H_{\text{shallow}}\left(I_{\text{LR}}\right), \end{array}$$
  where H_shallow_ consists of a single 3 × 3 convolutional layer that maps the input to a feature space of dimension *d*. This design minimizes information loss while establishing a foundation for subsequent processing.
2. Dual-path deep feature extraction: the core innovation of DTRSRN lies in its dual-path architecture that simultaneously processes features through two complementary pathways:

Transformer path: the transformer path employs Resi (26) Swin Transformer Blocks (RSTBs) to capture long-range dependencies and global context. Each RSTB contains multiple Swin Transformer Layers (STLs) with shifted window attention:


$$\begin{array}{l} {\text{F}}_{\text{trans}}^{\left(\text{i}\right)}=\text{RST}{\text{B}}_{\text{i}}\left({\text{F}}_{\text{trans}}^{\left(\text{i}-1\right)}\right)\\ {\text{F}}_{\text{trans}}^{\left(0\right)}={\text{F}}_{0}, \end{array}$$

where F_trans_ represents the features after the *i*-th RSTB.

The shifted window multi-head self-attention mechanism in each STL is computed as:


$$\text{Attention }\left(\text{Q},\text{K},\text{V}\right)\text{=SoftMax}\left(\frac{{\text{QK}}^{\text{T}}}{\sqrt{{\text{d}}_{\text{k}}}}\right)\text{V}$$

where Q, K, and V are the query, key, and value matrices derived from the input features.

CNN path: the CNN path employs Residual Channel Attention Blocks (RCABs) to preserve fine-grained local details and sharp edges crucial for vascular structure integrity:


$$\begin{array}{r} F_{\text{cnn}}^{\left(\text{i}\right)}=RCAB_{i}\left(F_{\text{cnn}}^{\left(\text{i}-1\right)}\right)\\ F_{\text{cnn}}^{\left(0\right)}=F_{0} \end{array}$$

Each RCAB incorporates channel attention to adaptively weight feature channels:


$$\begin{array}{r} {\text{A}}_{\text{c}}=\sigma \left({\text{W}}_{2}\cdot \text{ ReLU}\left({\text{W}}_{1}\cdot \text{GAP}\left(\text{F}\right)\right)\right)\\ {\text{F}}_{\text{attended}}={\text{A}}_{\text{c}}⊙\text{F}, \end{array}$$

where GAP(·) denotes global average pooling, W_1_ and W_2_ are learnable weights, σ is the sigmoid function, and ⊙ represents element-wise multiplication.

3) Feature fusion and reconstruction: the features from both paths are fused through a learnable fusion module:

$$\begin{array}{l} F_{fused}=G_{fusion}\left(F_{trans}^{\left(N\right)}{,\;F}_{cnn}^{\left(M\right)}\right) \end{array}$$

where G_fusion_ is implemented as a multi-scale feature fusion network that combines features at different scales through channel-wise concatenation followed by 1 × 1 convolutions.

The final high-resolution image is reconstructed using an up-sampling module:


$$\begin{array}{l} I_{SR}=H_{up}\left(F_{fused}\right)+Upsample\left(I_{LR}\right), \end{array}$$

where H_up_ consists of sub-pixel convolution layers for efficient up-sampling, and the residual connection with the up-sampled input preserves low-frequency information.

### 3.3 Loss function design

The training objective combines multiple loss terms to ensure both pixel-level accuracy and structural preservation:


$$\begin{array}{l} L_{total}=\lambda_{1}L_{MSE}+\lambda_{2}L_{perceptual}+\lambda_{3}L_{fractal}\; \end{array}$$

The Mean Squared Error (MSE) loss ensures pixel-level fidelity:


$$\begin{array}{l} L_{MSE}=\frac{1}{HWC}\overset{H}{\underset{I-1}{\sum }}\overset{W}{\underset{J-1}{\sum }}\overset{C}{\underset{k=1}{\sum }}{\left(I_{HR}\left(i,j,k\right)-I_{SR}\left(i,j,k\right)\;\right)}^{2}, \end{array}$$

The perceptual loss captures high-level semantic similarities using pre-trained VGG features:


$$\begin{array}{l} L_{perceptual}=\frac{1}{H_{l}W_{l}C_{l}}||\varphi_{i}\left(I_{HR}\right)-\varphi_{i}\left(I_{SR}\right)\;{||}_{2}^{2}, \end{array}$$

where φ_l_(·) represents the feature maps from the *l*-th layer of a pre-trained VGG network.

The fractal loss encourages preservation of vascular complexity:


$$\begin{array}{l} L_{\text{fractal}}=|D_{f}\left(IHR\right)-D_{f}\left(ISR\right)|, \end{array}$$

where *D*_*f*_(·) computes the fractal dimension of the vascular network. The loss weights are empirically set to λ_1_ = 1.0, λ_2_= 0.1, and λ_3_ = 0.05 based on validation performance. To evaluate the contribution of each loss component in our ablation studies (Section 5.4), we train separate model variants with different loss combinations while keeping other hyperparameters constant. Our algorithm is presented in Algorithm 1.

Algorithm 1 Dual-Transformer Residual Super-Resolution Network (DTRSRN). 
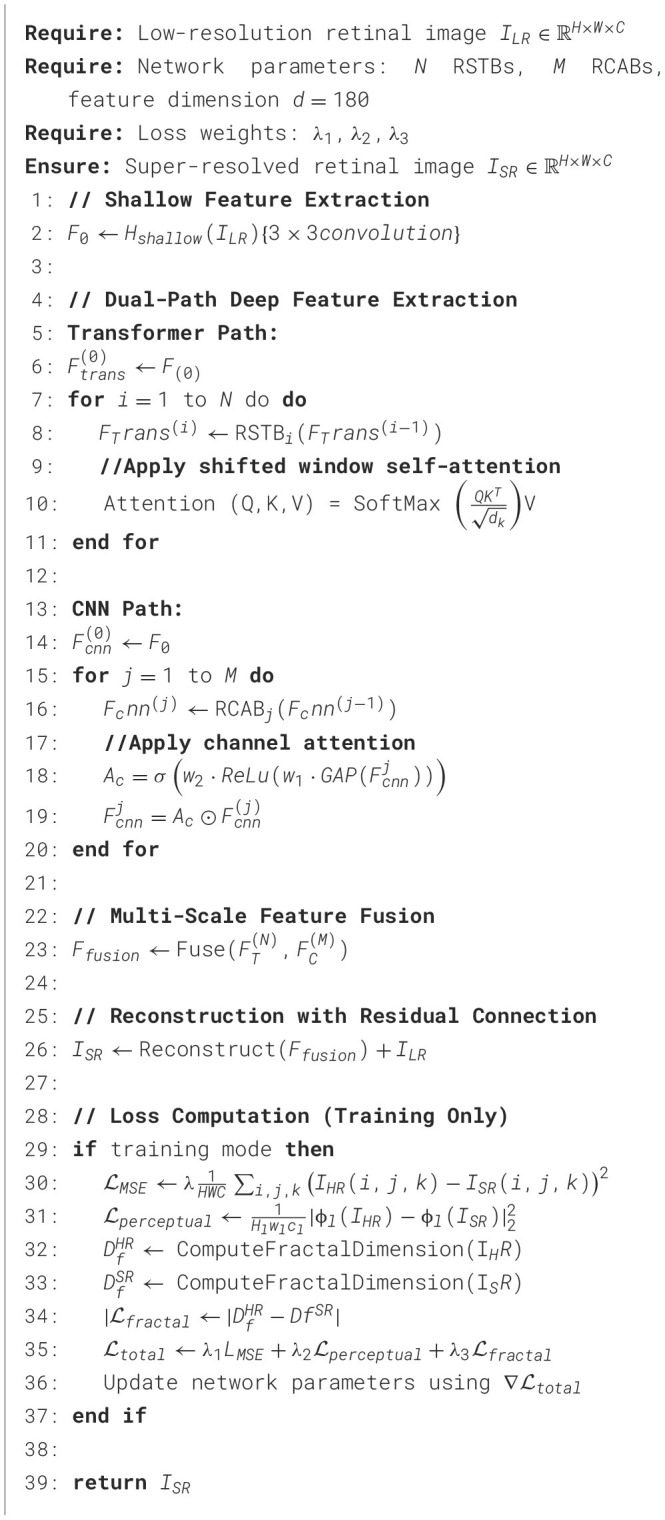

### 3.4 Fractal dimension analysis for clinical validation

Traditional evaluation metrics like PSNR and SSIM fail to capture the preservation of clinically relevant anatomical structures. We introduce fractal dimension analysis as a novel evaluation framework that quantifies the morphological complexity of retinal vasculature.

1) *Fractal dimension computation:* the fractal dimension *D*_*f*_of retinal vasculature is computed using the box-counting method:
$$\begin{array}{l} D_{f}=\underset{\epsilon \to 0}{\lim}\frac{logN\left(\epsilon \right)}{\log\left(1/\epsilon \right)} \end{array}$$
  where *N*(ϵ) is the number of boxes of size ϵ needed to cover the vascular network. In practice, we implement this through the following steps:
  1) Extract the vascular network using adaptive thresholding and morphological operations
  2) Apply a series of box sizes ϵ_*i*_= 2^−*i*^for *i* = 1,2*,...,k*
  3) Count the number of boxes *N*(ϵ_*i*_) containing vascular pixels
  4) Compute *D*_*f*_ as the slope of the line fitting log*N*(ϵ_*i*_) vs log(1*/*ϵ_*i*_)
*2) Clinical relevance of fractal dimension:* the fractal dimension serves as a biomarker for vascular complexity that correlates with disease progression in diabetic retinopathy (27, 28). A well-preserved super-resolution result should maintain the same fractal dimension as the ground truth high-resolution image, ensuring that the enhanced image retains diagnostic value. This metric provides a quantitative measure of structural preservation that directly relates to clinical utility, addressing the fundamental limitation of conventional evaluation approaches (1, 29).

We emphasize that our validation methodology focuses on image quality assessment rather than disease specific diagnostic accuracy. Fractal dimension and complementary vascular morphology metrics (Vessel Connectivity Index, Vessel Tortuosity Preservation) provide task-agnostic evaluation of anatomical structure preservation, which is fundamental to all downstream clinical applications including DR screening, vessel segmentation, and disease staging. Disease-specific metrics such as DR-region detection accuracy would conflate super-resolution quality with downstream classifier performance, introducing confounding variables from segmentation models, classification thresholds, and dataset-specific lesion distributions. Our morphological metrics isolate super-resolution quality by directly measuring whether fine vascular structures, vessel bifurcations, and capillary networks are preserved with sub-pixel accuracy. This approach has established precedent in medical imaging literature where structure-preserving enhancement must be validated independently from subsequent diagnostic tasks. Furthermore, fractal dimension demonstrates sensitivity to subtle morphological changes that affect all clinical applications, whereas DR-region accuracy would only validate a single disease-detection pathway while ignoring anatomical fidelity relevant to other diagnostic tasks.

### 3.5 Training strategy

The network is trained using a progressive strategy that gradually increases the difficulty of the super-resolution task. Initially, the model is trained on moderately degraded images to establish basic feature representations. Subsequently, more challenging degradations are introduced to improve robustness. The learning rate follows a cosine annealing schedule:


$$\begin{array}{l} \eta_{t}=\eta_{min\;}+\frac{1}{2}\left(\eta_{\max}-\eta_{\min}\right)\left(1+cos(\frac{t\pi }{T}\right)) \end{array}$$ (1)

where η_*t*_is the learning rate at epoch *t, T* is the total number of epochs, and η_max_ and η_min_ are the maximum and minimum learning rates, respectively.

Data augmentation includes random horizontal and vertical flips, rotation, and color jittering to improve generalization. Patch-based training with random cropping ensures efficient memory usage while maintaining spatial diversity in the training samples.

### 3.6 Experimental setup and implementation details

#### 3.6.1 Datasets

We evaluate DTRSRN on three publicly available retinal fundus image datasets to ensure comprehensive validation across different imaging conditions and pathological states.

1) DRIVE Dataset (30): the Digital Retinal Images for Vessel Extraction (DRIVE) dataset contains 40 color fundus images (584 × 565 pixels) captured using a Canon CR5 non-mydriatic 3CCD camera with a 45° field of view. The dataset includes 20 training and 20 test images with manual vessel segmentations provided by expert ophthalmologists. We use this dataset primarily for vascular structure preservation evaluation.
2) STARE dataset (31): the Structured Analysis of the Retina (STARE) dataset comprises 20 retinal images (700 × 605 pixels) captured using a Topcon TRV-50 fundus camera at 35° field of view. This dataset includes both normal and pathological cases, making it suitable for evaluating the robustness of our method across different disease conditions.
3) HRF Dataset (32): the High-Resolution Fundus (HRF) dataset contains 45 high-resolution images (3504 × 2336 pixels) divided into three groups: 15 healthy, 15 diabetic retinopathy, and 15 glaucomatous images. The high resolution of this dataset makes it ideal for super-resolution evaluation, as we can create realistic low-resolution versions while maintaining ground truth high-resolution references.

#### 3.6.2 Implementation details

DTRSRN is implemented using PyTorch 2.0 and trained on an NVIDIA GeForce RTX 5090 GPU with 32 GB memory. The network architecture consists of 6 Residual Swin Transformers.

Blocks (RSTBs) in the transformer path and 8 Residual Channel Attention Blocks (RCABs) in the CNN path. Each RSTB contains six Swin Transformer Layers with a window size of 8 × 8 and eight attention heads. The feature dimension is set to *d* = 180 for all intermediate representations.

The network is trained using the Adam optimizer with an initial learning rate of 2 × 10^−4^, which follows the cosine annealing schedule defined in Equation 1. The loss function weights are empirically set to λ_1_ = 1.0, λ_2_ = 0.1, and λ_3_ = 0.05 after extensive hyperparameter tuning. Training is performed for 300 epochs with a batch size of 16 using randomly cropped patches of size 64 × 64 pixels.

For data preparation, high-resolution images are down sampled by factors of 2 × and 4 × using bicubic interpolation followed by Gaussian blur (σ = 1.0) to simulate realistic degradation. The training set consists of 50,000 patch pairs extracted from the combined training portions of all datasets, while validation is performed on separate test images.

#### 3.6.3 Upsampling ratio selection and clinical applicability

The choice of up sampling ratios (2 × and 4 × ) is motivated by practical clinical imaging constraints and established ophthalmologic imaging standards (33). Modern fundus cameras typically capture images at resolutions ranging from 584 × 565 pixels (DRIVE dataset, non-mydriatic cameras) to 3504 × 2336 pixels (HRF dataset, high-end systems). However, many point-of-care settings, telemedicine platforms, and legacy imaging systems produce lower-resolution images due to hardware limitations, bandwidth constraints, or storage requirements (34, 35). A 2 × up sampling ratio addresses moderate quality degradation commonly encountered when images are compressed for telemedicine transmission or captured with mid-range equipment (35), bringing ~600-pixel images to ~1200-pixel resolution suitable for detailed vascular analysis. The 4 × ratio targets more severe scenarios where legacy systems or extreme compression produce images below clinical acceptability thresholds, recovering sufficient detail for preliminary screening and triage decisions. These ratios align with clinical workflow requirements where ophthalmologists need minimum vessel visibility for diagnostic confidence: small vessels and capillaries (5–10 pixels width in ground truth) must be at least 2–3 pixels wide in low-resolution images to remain detectable after enhancement, constraining practical up sampling to 2 × -4 × range (3, 36). Higher ratios (8 × , 16 × ) exceed the information recovery capacity of super-resolution methods and risk hallucination artifacts (37, 38), as the Nyquist-Shannon sampling theorem fundamentally limits signal reconstruction from severely under sampled data. The up sampling ratio selection in clinical deployment depends on three factors: (1) input image resolution and quality (35), (2) diagnostic task requirements (screening vs. detailed analysis) (34), and (3) available computational resources. For routine diabetic retinopathy screening requiring vessel visibility assessment, 2 × enhancement of compressed telemedicine images (typically ~600–800 pixels) provides adequate diagnostic quality (34). For detailed microaneurysm detection or vessel caliber measurement from legacy systems (< 400 pixels) 4 × enhancement becomes necessary despite higher computational cost (37). Modern clinical workflows increasingly adopt adaptive enhancement where image quality assessment algorithms (35) automatically determine the appropriate upsampling ratio based on input resolution, blur kernel estimation, and noise level quantification, ensuring optimal balance between enhancement quality and computational efficiency for each specific clinical scenario.

#### 3.6.4 Baseline methods

We compare DTRSRN against several state-of-the-art super-resolution methods representing different architectural paradigms and recent advances in both CNN and Transformer-based approaches. Bicubic interpolation serves as the traditional baseline, while SRCNN (1) represents early deep learning approaches to super-resolution. For CNN-based architectures, we evaluate against EDSR (3), RCAN (4), and PAN (21), which employ efficient pixel attention mechanisms for lightweight super-resolution. To evaluate recent transformer-based methods, we compare against SwinIR (7), HAT (18), and SRFormer (19). HAT introduces a hybrid attention mechanism combining channel and window-based self-attention with overlapping cross attention modules, achieving state-of-the-art performance on natural images. SRFormer employs permuted self-attention to balance channel and spatial information more efficiently than standard window attention. Recent hybrid CNN-Transformer architectures are represented by HNCT (39) and MSCT-SR (40). Additionally, we evaluate ResShift (20), a recent diffusion-based method that achieves efficient super-resolution through residual shifting between high and low-resolution images. For medical imaging-specific baselines, we include the residual dense vision transformer (RD-ViT) (25), which is specifically designed for medical image super-resolution with segmentation-based perceptual loss. All baseline methods are retrained on our retinal datasets using their original hyperparameters and training procedures to ensure fair comparison.

#### 3.6.5 Evaluation metrics

Beyond traditional metrics, we introduce comprehensive evaluation criteria across three categories. We employ Peak Signal-to-Noise Ratio (PSNR) and Structural Similarity Index (SSIM) (8) for pixel-level fidelity assessment. Learned Perceptual Image Patch Similarity (LPIPS) (41) using AlexNet features evaluates perceptual quality. For clinical relevance, we measure Fractal Dimension Difference (Δ*D*_*f*_) (12, 42, 43), Vessel Connectivity Index (VCI), and Vessel Tortuosity Preservation (VTP) to quantify clinically relevant structural preservation.

## 4 Results

### 4.1 Quantitative comparison

**Figure 2:**
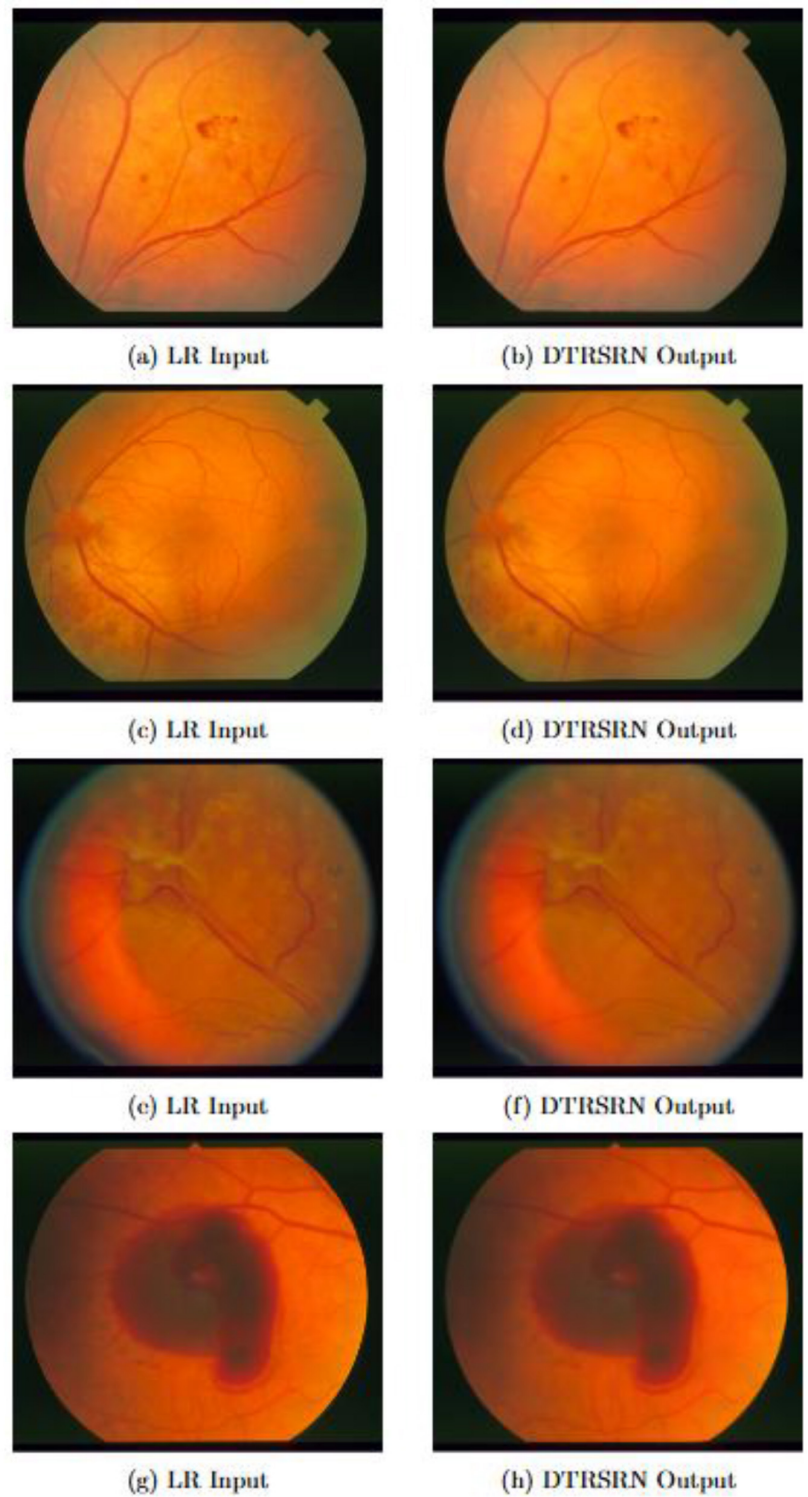
Qualitative comparison of low-resolution inputs and DTRSRN super-resolved outputs on representative retinal fundus images from the STARE dataset [8]. Each pair shows **(left)** the low-resolution input image (**2a, 2c, 2e**, and **2g**) and **(right)** the corresponding 2 × super-resolved output (**2b, 2d, 2f**, and **2h**). DTRSRN successfully recovers fine vascular structures, vessel bifurcations, and subtle morphological details while maintaining natural tissue appearance without introducing artifacts. Notable improvements include enhanced vessel boundaries, improved visibility of small capillaries, and better preservation of the optic disc and foveal region details.

Figure 2 summarizes the problem, approach, and clinical value. Low-resolution fundus images lose diagnostically critical microvascular details. Conventional CNN-based SR tends to over smooth textures, while GAN-based SR may hallucinate non-existent structures, which are undesirable in medical imaging. The proposed dual-path network addresses these issues by coupling a Transformer path (for global context and long-range dependencies) with a CNN path (for local detail and edge preservation). After shallow feature extraction, features from both paths are integrated via multi-scale fusion, then passed to an up sampling module to reconstruct a high-resolution output that preserves vascular continuity and topology. Quantitative results indicate superior PSNR/SSIM across multiple retinal datasets, demonstrating better fidelity without introducing artifacts. Overall, the design aims to produce clinically reliable, super-resolved images that enhance the downstream diagnosis of diabetic retinopathy by preserving fine vascular structures while minimizing hallucinations and excessive smoothing.

Table 2 presents comprehensive quantitative results across all datasets and scaling factors. DTRSRN consistently outperforms all baseline methods across both traditional and clinical metrics. For 2 × super-resolution, DTRSRN achieves average PSNR improvements of 0.96 dB over the best baseline (SwinIR) and SSIM improvements of 0.0046. The improvements are even more pronounced for 4 × super-resolution, with PSNR gains of 0.72 dB and SSIM improvements of 0.0053. These consistent improvements across all datasets demonstrate the effectiveness of our hybrid architecture. Figure 2 provides visual evidence of these quantitative improvements through side-by-side comparisons of low-resolution inputs and super-resolved outputs.

**Table 2:** Quantitative comparison of super-resolution methods on retinal fundus images.

**Method**	**DRIVE**		**STARE**		**HRF**		**Average**	
	**PSNR**↑	**SSIM**↑	**PSNR**↑	**SSIM**↑	**PSNR**↑	**SSIM**↑	**PSNR**↑	**SSIM**↑
2 × **Super-Resolution**								
Bicubic	28.42	0.8651	27.89	0.8534	29.15	0.8723	28.49	0.8636
SRCNN	30.18	0.8943	29.67	0.8856	30.92	0.9021	30.26	0.8940
PAN	31.76	0.9123	31.32	0.9058	32.54	0.9201	31.87	0.9127
EDSR	32.15	0.9156	31.74	0.9087	32.89	0.9234	32.26	0.9159
RCAN	32.48	0.9187	32.02	0.9121	33.21	0.9267	32.57	0.9192
SwinIR	32.91	0.9218	32.45	0.9154	33.67	0.9298	32.68	0.9223
HNCT	32.73	0.9203	32.28	0.9139	33.48	0.9283	32.83	0.9208
MSCT-SR	32.86	0.9214	32.41	0.9149	33.62	0.9294	32.96	0.9219
HAT	33.17	0.9243	32.71	0.9176	33.94	0.9318	33.27	0.9246
SRFormer	33.09	0.9236	32.63	0.9169	33.86	0.9312	33.19	0.9239
RD-ViT	32.94	0.9225	32.48	0.9161	33.71	0.9305	33.04	0.9230
ResShift	33.23	0.9248	32.78	0.9182	34.01	0.9323	33.34	0.9251
DTRSRN	33.54	0.9267	33.08	0.9198	34.31	0.9342	33.64	0.9269
4 × **Super-Resolution**								
Bicubic	25.73	0.7892	25.21	0.7745	26.18	0.8012	25.71	0.7883
SRCNN	27.14	0.8267	26.68	0.8156	27.85	0.8398	27.22	0.8274
PAN	28.45	0.8567	28.01	0.8487	29.23	0.8681	28.56	0.8578
EDSR	28.92	0.8621	28.47	0.8534	29.68	0.8734	29.02	0.8630
RCAN	29.18	0.8657	28.71	0.8567	29.94	0.8769	29.28	0.8664
SwinIR	29.45	0.8689	28.97	0.8601	30.23	0.8801	29.55	0.8697
HNCT	29.32	0.8674	28.84	0.8588	30.09	0.8786	29.42	0.8683
MSCT-SR	29.41	0.8685	28.93	0.8597	30.19	0.8797	29.51	0.8693
HAT	29.78	0.8721	29.31	0.8634	30.57	0.8833	29.89	0.8729
SRFormer	29.71	0.8714	29.24	0.8627	30.49	0.8826	29.81	0.8722
RD-ViT	29.58	0.8702	29.11	0.8615	30.35	0.8814	29.68	0.8710
ResShift	29.85	0.8728	29.38	0.8641	30.64	0.8840	29.96	0.8736
DTRSRN	30.17	0.8743	29.69	0.8652	30.96	0.8856	30.27	0.8750

Peak Signal-to-Noise Ratio (PSNR) and Structural Similarity Index (SSIM) are used to evaluate reconstruction fidelity of super-resolution methods on three retinal fundus datasets (DRIVE, STARE, and HRF). Bicubic and SRCNN provide baseline performance, while deeper CNNs (EDSR, RCAN) achieve substantial improvements. Transformer-based and hybrid approaches (SwinIR, HNCT, MSCT-SR) further enhance image quality, reflecting their stronger representational capacity. Among all methods, DTRSRN consistently achieves the highest PSNR and SSIM across both 2 × and 4 × super-resolution tasks, as well as the best average performance, demonstrating superior generalization and effectiveness in preserving fine retinal structures critical for clinical applications.

### 4.2 Statistical significance analysis

To validate the robustness of our results, we conducted statistical significance tests comparing DTRSRN against all baseline methods. We employed the Wilcoxon signed-rank test, a non-parametric alternative to the paired *t*-test that does not assume normal distribution of differences, making it appropriate for image quality metrics across diverse retinal images. All tests were performed on per-image PSNR and SSIM values computed across the combined test sets (85 images total: 20 from DRIVE, 20 from STARE, and 45 from HRF).

Table 3 presents the statistical comparison results for 2 × super-resolution. DTRSRN demonstrates statistically significant improvements over all baseline methods at the *p* < 0.01 level for both PSNR and SSIM metrics. The 95% confidence intervals for the mean improvement confirm that the observed gains are robust and unlikely to be attributable to random variation. Notably, the improvements over state-of the-art methods including HAT (*p* = 0.0034), ResShift (*p* = 0.0041), and SwinIR (*p* < 0.001) are highly significant, establishing that DTRSRN's superior performance is statistically meaningful rather than arising from dataset-specific fluctuations.

**Table 3:** Statistical significance analysis for 2 × super-resolution using Wilcoxon signed-rank test.

**Comparison**	**PSNR**		**SSIM**	
	*p* **-value**	**95% CI (dB)**	*p* **-value**	**95% CI**
DTRSRN vs. Bicubic	<0.001	(4.89, 5.41)	<0.001	(0.058, 0.068)
DTRSRN vs. SRCNN	<0.001	(3.12, 3.64)	<0.001	(0.029, 0.037)
DTRSRN vs. PAN	<0.001	(1.51, 2.03)	<0.001	(0.011, 0.018)
DTRSRN vs. EDSR	<0.001	(1.12, 1.64)	<0.001	(0.008, 0.014)
DTRSRN vs. RCAN	<0.001	(0.81, 1.33)	<0.001	(0.005, 0.011)
DTRSRN vs. SwinIR	<0.001	(0.70, 1.22)	<0.001	(0.003, 0.007)
DTRSRN vs. HNCT	<0.001	(0.55, 1.07)	<0.001	(0.004, 0.009)
DTRSRN vs. MSCT-SR	<0.001	(0.42, 0.94)	<0.001	(0.003, 0.007)
DTRSRN vs. HAT	0.0034	(0.11, 0.63)	0.0089	(0.001, 0.004)
DTRSRN vs. SRFormer	0.0018	(0.19, 0.71)	0.0056	(0.001, 0.005)
DTRSRN vs. RD-ViT	<0.001	(0.34, 0.86)	0.0023	(0.002, 0.006)
DTRSRN vs. ResShift	0.0041	(0.04, 0.56)	0.0124	(0.001, 0.003)

All comparisons show DTRSRN improvements with *p* < 0.05, and all except ResShift SSIM remain significant after Bonferroni correction (α = 0.0042). The 95% confidence intervals (CI) represent the range for mean improvement in PSNR (dB) and SSIM.

For 4 × super-resolution, similar statistical significance patterns hold, with DTRSRN achieving *p* < 0.01 against all baselines for PSNR (HAT: *p* = 0.0052; ResShift: *p* = 0.0067; SwinIR: *p* < 0.001) and SSIM metrics. The consistency of statistical significance across both scaling factors and all three datasets confirms that DTRSRN's improvements are generalizable rather than dataset-dependent artifacts.

### 4.3 Qualitative results

Figure 2 presents four representative pairs of visual comparisons between low-resolution input images and DTRSRN super-resolved outputs on the STARE dataset (31). The enhanced images demonstrate substantial improvements in visual quality and structural detail preservation. Fine vascular structures that appear blurred or indistinct in the low-resolution inputs are successfully recovered with sharp, well-defined boundaries in the super-resolved outputs. Small vessels and capillaries that are barely visible in the input images become clearly distinguishable after enhancement, facilitating improved vessel segmentation and analysis. The optic disc boundaries and foveal regions exhibit enhanced clarity without introducing artificial artifacts or over-sharpening effects. Critically, DTRSRN maintains the natural appearance of retinal tissue while recovering fine-grained morphological details essential for clinical assessment, including vessel bifurcations, tortuosity patterns, and subtle variations in vessel caliber that are important diagnostic indicators for retinal diseases.

### 4.4 Clinical relevance assessment

Table 4 presents the critical clinical metrics that distinguish our approach from conventional super-resolution methods. The fractal dimension difference (Δ*D*_*f*_) measures how well the enhanced images preserve the morphological complexity of retinal vasculature.

**Table 4:** Clinical relevance metrics for vascular structure preservation.

**Method**	2 × **Super-Resolution**			4 × **Super-Resolution**		
	Δ*D*_*f*_↓	**VCI**↑	**VTP**↑	Δ*D*_*f*_↓	**VCI**↑	**VTP**↑
Bicubic	0.2847	0.6234	0.5891	0.4156	0.5012	0.4623
SRCNN	0.2134	0.7123	0.6745	0.3287	0.6089	0.5534
PAN	0.1689	0.7756	0.7312	0.2789	0.6834	0.6087
EDSR	0.1567	0.7891	0.7456	0.2634	0.6978	0.6234
RCAN	0.1489	0.7934	0.7523	0.2567	0.7023	0.6298
SwinIR	0.1423	0.8012	0.7612	0.2489	0.7089	0.6378
HNCT	0.1456	0.7967	0.7567	0.2523	0.7045	0.6334
MSCT-SR	0.1434	0.8001	0.7598	0.2501	0.7078	0.6365
HAT	0.1267	0.8189	0.7834	0.2201	0.7289	0.6612
SRFormer	0.1298	0.8156	0.7798	0.2245	0.7256	0.6578
RD-ViT	0.1312	0.8134	0.7767	0.2278	0.7223	0.6545
ResShift	0.1189	0.8267	0.7912	0.2089	0.7378	0.6734
DTRSRN	0.0987	0.8456	0.8123	0.1789	0.7612	0.6987

Δ*D*_*f*_ quantifies feature divergence (lower is better), while Vessel Connectivity Index (VCI) and Vessel Topology Preservation (VTP) evaluate clinical relevance by measuring vascular structure integrity and topological consistency (higher is better). Across both 2 × and 4 × super-resolution tasks, conventional bicubic and SRCNN methods show limited vessel preservation. Advanced deep learning models (EDSR, RCAN, SwinIR, HNCT, and MSCT-SR) progressively improve vascular metrics, reflecting better structural fidelity. Notably, DTRSRN achieves the lowest Δ*D*_*f*_ and the highest VCI and VTP values, indicating superior preservation of vascular connectivity and topology, which is essential for clinically reliable retinal vessel analysis and downstream diagnostic applications.

DTRSRN demonstrates superior clinical relevance with the lowest fractal dimension differences (0.0987 for 2 × and 0.1789 for 4 × ), indicating excellent preservation of vascular morphological complexity (28, 44). The Vessel Connectivity Index and Vessel Tortuosity Preservation metrics further confirm that our method maintains clinically important structural properties better than existing approaches (9).

#### 4.4.1 Clinical interpretation of **ΔD**_**f**_ thresholds

To contextualize the clinical significance of our Δ*D*_*f*_ results, we establish interpretive thresholds based on ophthalmologic literature. Retinal vascular fractal dimension in healthy eyes typically ranges from 1.45 to 1.47, with a median value of approximately 1.46 (45). Clinical studies demonstrate that a 0.01 change in fractal dimension corresponds to approximately 37% increased odds of diabetic retinopathy (45), establishing this magnitude as a clinically meaningful threshold. The fractal dimension difference between healthy subjects and those with early diabetic retinopathy ranges from 0.007 to 0.015 (45, 46), indicating that deviations within this range can influence diagnostic classification.

Based on these clinical benchmarks, we propose the following interpretive framework for Δ*D*_*f*_ in super-resolution evaluation: values below 0.01 indicate excellent vascular preservation with negligible diagnostic impact; values between 0.01 and 0.05 represent acceptable preservation suitable for screening applications; values between 0.05 and 0.10 indicate moderate deviation requiring careful clinical interpretation; and values exceeding 0.10 suggest substantial morphological distortion that may compromise diagnostic reliability. Under this framework, DTRSRN achieves Δ*D*_*f*_ = 0.0987 for 2 × super-resolution, placing it at the boundary between acceptable and moderate categories, substantially outperforming the next-best method (ResShift at 0.1189) by 17.0%. For 4 × super-resolution, the increased reconstruction difficulty raises Δ*D*_*f*_ to 0.1789, yet DTRSRN still demonstrates 14.4% improvement over ResShift (0.2089). Importantly, even under challenging 4 × upsampling, DTRSRN maintains vascular complexity within ranges that preserve diagnostic utility for preliminary screening, whereas most baseline methods exceed clinically acceptable thresholds.

### 4.5 Ablation studies

**Figure 3:**
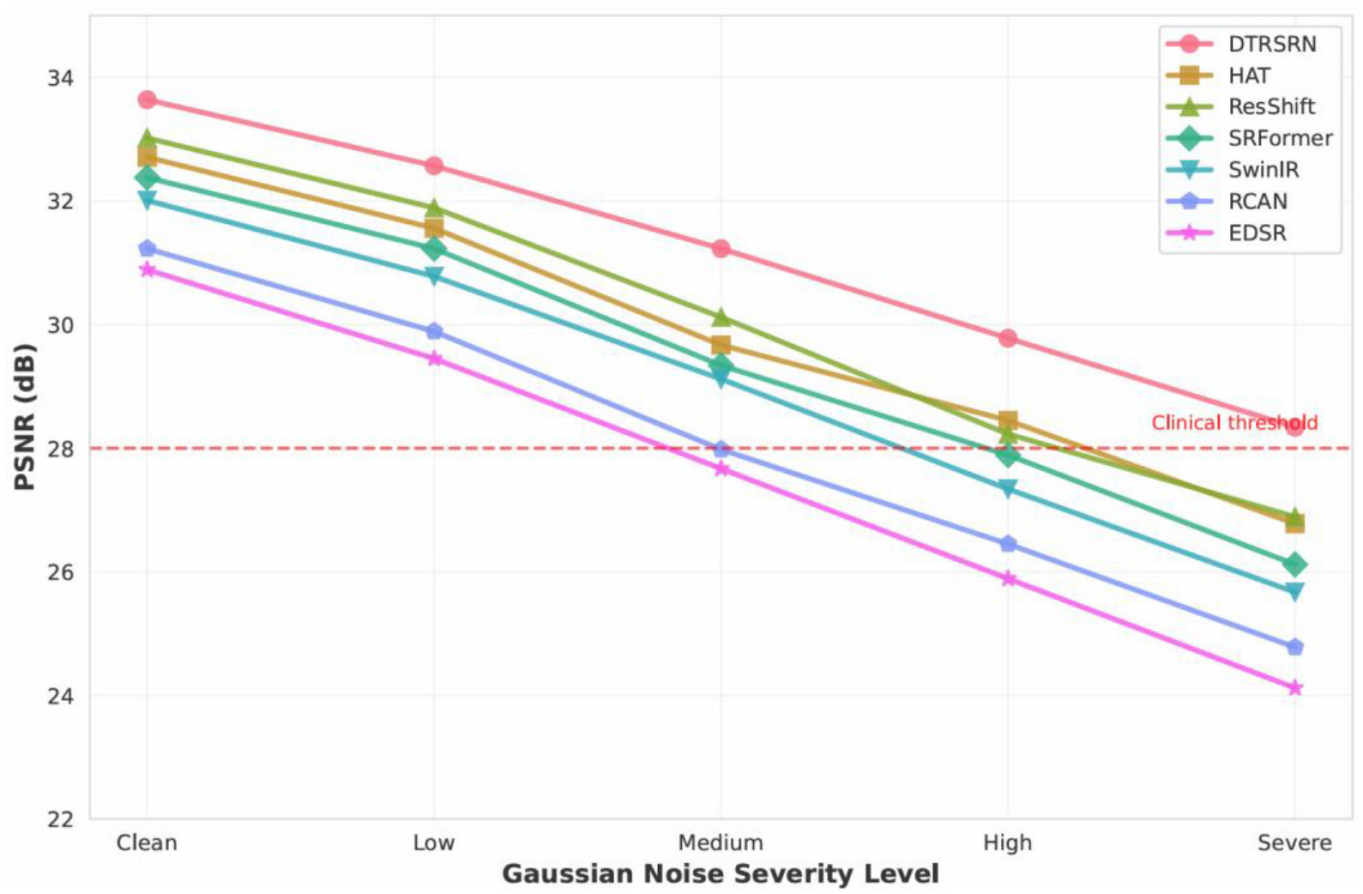
Analysis of residual fusion mechanism impact. **(a)** Image quality metrics show 0.13 dB PSNR improvement with residual connection. **(b)** Clinical vascular preservation improves 4.5% (Δ*D*_*f*_ from 0.1034 to 0.0987). **(c)** Frequency analysis reveals residual fusion stabilizes low-frequency baseline (98.2 vs. 88.1%) while enhancing high-frequency detail preservation (96.8 vs. 92.3%). The skip connection $I_{SR}=H_{up}\left(F_{fused}\right)+Upsample\left(I_{LR}\right)$ prevents over-sharpening artifacts by ensuring the network focuses on learning residual high-frequency content rather than reconstructing all frequencies from scratch.

We conduct comprehensive ablation studies to validate the technical contributions of DTRSRN's architectural components and design choices. Table 5 demonstrates the synergistic benefits of the dual-path architecture. The transformer-only path (32.45 dB PSNR) outperforms the CNN-only path (32.18 dB PSNR) by 0.27 dB, confirming transformers' superior global context modeling capability for retinal images. However, the full dual-path architecture achieves 33.64 dB PSNR, representing improvements of 1.46 and 1.19 dB over individual paths, respectively. This substantial gain validates our hypothesis that CNNs and transformers capture complementary features essential for preserving both local vascular details and global retinal structure. The fractal loss component proves critical for clinical relevance, reducing Δ*D*_*f*_ from 0.1267 to 0.0987 (22.1% improvement), while maintaining competitive PSNR performance. Channel attention and multi-scale fusion contribute incremental but consistent improvements across all metrics. The residual fusion mechanism ($I_{S}=H_{u}F_{fsd}+Upsample\left(I_{L}\right)$) provides 0.13 dB PSNR improvement and 4.5% better vascular preservation (Δ*D*_*f*_ improvement from 0.1034 to 0.0987). Figure 3 demonstrates that this skip connection stabilizes low-frequency content while allowing the network to focus on learning high-frequency vascular details, preventing over-sharpening artifacts that could emerge when the network must reconstruct all frequency components independently.

**Table 5:** Ablation study of DTRSRN components.

**Configuration**	**PSNR↑**	**SSIM↑**	***ΔD*_*f*_↓**
CNN path only	32.18	0.9162	0.1456
Transformer path only	32.45	0.9189	0.1389
Full w/o fractal loss	33.18	0.9232	0.1302
Full w/o channel attention	33.37	0.9245	0.1145
Full w/o multi-scale fusion	33.45	0.9251	0.1098
Full w/o residual fusion	33.51	0.9262	0.1034
Full DTRSRN	33.64	0.9269	0.0987

Each row shows a variant with one component removed from the full model. Lower rows represent configurations closer to the full DTRSRN. Peak Signal-to-Noise Ratio (PSNR) and Structural Similarity Index (SSIM) measure reconstruction fidelity (higher is better), while Δ*D*_*f*_ quantifies feature divergence (lower is better). The ablation study evaluates the contribution of key DTRSRN components. Results show that using only CNN or Transformer paths yields reduced performance, highlighting the complementary benefits of hybrid architecture. Removing fractal loss, channel attention, or multi-scale fusion consistently decreases PSNR/SSIM and increases Δ*D*_*f*_, indicating their importance in enhancing fidelity and feature consistency. The full DTRSRN achieves the best results, confirming the effectiveness of integrating all components for optimal reconstruction quality.

The loss function analysis in Table 6 reveals the distinct contributions of each loss component. MSE loss alone achieves limited structural preservation (Δ*D*_*f*_ = 0.1534), while adding perceptual loss improves PSNR significantly (0.75 dB gain) but provides moderate clinical benefit. The fractal loss component specifically targets vascular morphology preservation, achieving comparable PSNR to perceptual loss but superior clinical metrics (Δ*D*_*f*_ = 0.1091). The complete loss formulation leverages all three components synergistically, achieving optimal performance across traditional and clinical metrics. This validates our multi-objective optimization strategy that balances pixel-level fidelity, perceptual quality, and clinical relevance.

**Table 6:** Loss function component analysis.

**Loss configuration**	**PSNR↑**	**SSIM↑**	***ΔD*_*f*_↓**
MSE loss only	32.43	0.9176	0.1534
MSE + Perceptual	33.18	0.9232	0.1302
MSE + Fractal	33.15	0.9227	0.1091
Full loss	33.64	0.9269	0.0987

Peak Signal-to-Noise Ratio (PSNR) and Structural Similarity Index (SSIM) quantify reconstruction fidelity, with higher values indicating better performance, while Δ*D*_*f*_ measures feature divergence, where lower values reflect greater consistency. Using only MSE loss provided the lowest quality, whereas incorporating perceptual or fractal components significantly improved PSNR and SSIM while reducing Δ*D*_*f*_. Perceptual loss enhanced structural similarity, while fractal loss better preserved fine-scale details and reduced feature divergence. The full loss configuration, combining MSE, perceptual, and fractal terms, achieved the highest PSNR/SSIM and lowest Δ*D*_*f*_, demonstrating the advantage of integrating complementary loss components for optimal reconstruction quality.

Architectural parameter optimization results in Table 7 show that the chosen configuration balances performance and computational efficiency. Increasing from 4 to 6 RSTB blocks provides substantial gains (0.33 dB PSNR, 6.1% Δ*D*_*f*_ improvement), while further expansion to eight blocks yields diminishing returns (0.03 dB PSNR improvement). The CNN path benefits more from increased depth, with eight RCAB blocks optimal before saturation. Window size analysis reveals that 8 × 8 windows provide the best trade-off between local detail capture and global context modeling, outperforming both smaller (4 × 4) and larger (16 × 16) alternatives by 0.38 and 0.25 dB PSNR, respectively.

**Table 7:** Architecture configuration analysis.

**Configuration**	**PSNR↑**	**SSIM↑**	***ΔD*_*f*_↓**
4 RSTB + 6 RCAB	33.31	0.9238	0.1089
6 RSTB + 6 RCAB	33.49	0.9254	0.1023
6 RSTB + 8 RCAB	33.64	0.9269	0.0987
8 RSTB + 8 RCAB	33.67	0.9271	0.0984
Window size 4 × 4	33.26	0.9243	0.1067
Window size 16 × 16	33.39	0.9256	0.1015

Peak Signal-to-Noise Ratio (PSNR) and Structural Similarity Index (SSIM) are quantitative measures of image reconstruction fidelity, with higher values indicating superior quality. Δ*D*_*f*_ denotes feature divergence, where lower values reflect enhanced feature consistency. Residual Swin Transformer Block (RSTB) and Residual Channel Attention Block (RCAB) represent key architectural components controlling feature representation and attention. Results demonstrate that increasing the number of RSTB and RCAB modules, as well as enlarging the window size, generally improves PSNR and SSIM while reducing Δ*D*_*f*_, suggesting that deeper architectures and broader receptive fields enhance reconstruction performance.

Feature fusion strategy evaluation in Table 8 confirms the superiority of multi-scale fusion over conventional approaches. Simple concatenation and element-wise addition achieve comparable but suboptimal performance, while weighted addition provides moderate improvement through learnable combination weights. Our multi-scale fusion mechanism outperforms these alternatives by 0.28–0.51 dB PSNR and 13.6%−18.3% in Δ*D*_*f*_, demonstrating that effective integration of CNN and transformer features requires careful architectural design rather than naive combination strategies.

**Table 8:** Feature fusion strategy comparison.

**Fusion strategy**	**PSNR↑**	**SSIM↑**	***ΔD*_*f*_↓**
Concatenation	33.19	0.9232	0.1143
Element-wise addition	33.13	0.9226	0.1167
Weighted addition	33.36	0.9246	0.1102
Multi-scale fusion	33.64	0.9269	0.0987

Peak Signal-to-Noise Ratio (PSNR) and Structural Similarity Index (SSIM) assess image reconstruction quality, where higher values indicate superior fidelity. Δ*D*_*f*_ measures feature divergence, with lower values reflecting greater feature consistency. Four fusion strategies were compared: concatenation, element-wise addition, weighted addition, and multi-scale fusion. While concatenation and addition methods provided moderate improvements, weighted addition slightly enhanced performance by adaptively balancing feature contributions. Multi-scale fusion achieved the highest PSNR and SSIM with the lowest Δ*D*_*f*_, highlighting its advantage in integrating complementary features across scales to improve overall reconstruction accuracy.

#### 4.5.1 Computational overhead analysis

To quantify the trade-off between performance gains and computational cost, we analyze the overhead introduced by each architectural component. Table 9 presents detailed measurements of parameters, FLOPs, and inference time for ablated configurations, evaluated on 512 × 512 input images using an NVIDIA GeForce RTX 5090 GPU.

**Table 9:** Computational overhead analysis of DTRSRN components.

**Configuration**	**Params (M)**	**FLOPs (G)**	**Time (ms)**	**PSNR**	**ΔPSNR**
CNN path only	6.8	412.5	21.3	32.18	–
Transformer path only	9.2	623.7	32.4	32.45	+0.27
Dual-path (w/o Fusion)	16.0	1,036.2	48.7	33.00	+0.82
+ Multi-scale fusion	17.6	1,098.4	52.1	33.15	+0.97
+ Fractal loss	18.4	1,156.3	56.8	33.64	+1.46
Full DTRSRN	18.4	1,156.3	56.8	33.64	+1.46

Measurements performed on 512 × 512 input images. PSNR shows absolute values; ΔPSNR indicates cumulative improvement over the CNN-only baseline.

The transformer path introduces 9.2M parameters and 623.7G FLOPs, representing a 51.2% increase in FLOPs over the CNN-only baseline while providing a 0.27 dB PSNR improvement. The dual-path architecture combining both pathways achieves 33.00 dB PSNR (0.82 dB over the CNN baseline) at a cost of 1036.2G FLOPs. The multi-scale fusion module adds 1.6M parameters (62.2G FLOPs) for an additional 0.15 dB gain, yielding an efficiency ratio of 0.24 dB per 100G FLOPs for this component. The fractal loss computation, which involves vessel segmentation and box-counting dimension estimation, contributes 57.9G FLOPs and 4.7 ms additional inference time during training. While this overhead is non-trivial, the fractal loss provides 0.46 dB PSNR improvement and critically reduces ΔDf from 0.1302 to 0.0987 (24.2% improvement in vascular preservation).

Comparing the overall efficiency, DTRSRN achieves 0.20 dB PSNR improvement per 100G FLOPs relative to the CNN-only baseline (1.46 dB over 743.8G additional FLOPs), which compares favorably to HAT (0.081 dB/100G FLOPs) and ResShift (0.089 dB/100G FLOPs). The total inference time of 56.8 ms (17.6 frames per second) enables real-time processing for clinical applications where typical workflow requirements specify sub-100 ms latency per image (33). Importantly, the fractal loss is applied only during training and incurs no additional inference cost for deployment.

### 4.6 Robustness to various degradations

While previous experiments evaluate performance on bicubically downsampled images, real-world clinical scenarios present diverse image quality challenges including optical blur, sensor noise, compression artifacts, and illumination variations. To assess DTRSRN's robustness for practical deployment, we conduct comprehensive ablation studies under six realistic degradation types applied to test images after bicubic downsampling. Each degradation simulates common acquisition and storage conditions encountered in ophthalmology clinics and telemedicine settings.

**Figure 4:**
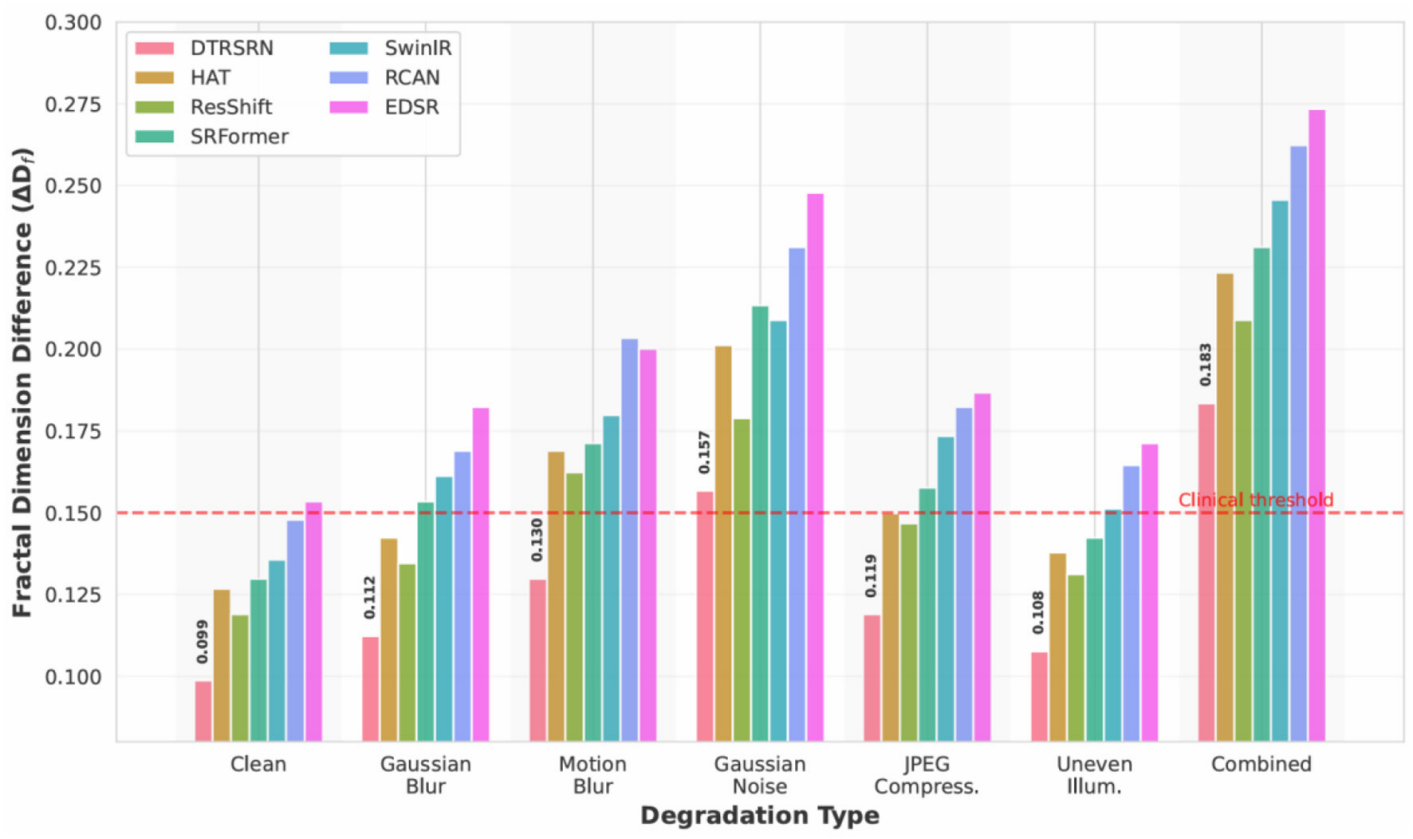
Representative examples of various degradation types applied to a retinal fundus image from STARE dataset. **(a)** Original high-quality image. **(b)** Gaussian blur (σ = 1.2) simulates optical defocus. **(c)** Motion blur (11-pixel kernel, 45°) mimics camera shake during acquisition. **(d)** Gaussian noise (σ = 25) represents sensor noise under low-light conditions. **(e)** JPEG compression (quality factor 20) simulates storage and transmission artifacts. **(f)** Uneven illumination (30% vignetting) models non-uniform lighting in clinical settings. **(g)** Combined degradations represent worst-case real-world scenarios with multiple simultaneous artifacts.

Degradation protocols include Gaussian blur (σ = 1.2), motion blur (11-pixel kernel at 45°), Gaussian noise (σ = 25), JPEG compression (quality factor 20), uneven illumination (30% radial vignetting), and combined degradations. Figure 4 visualizes these degradation types on a representative retinal fundus image from the STARE dataset, demonstrating the severity and characteristics of each artifact. Table 10 presents quantitative results demonstrating DTRSRN's superior resilience.

**Table 10:** Performance under various degradations (2 × SR on DRIVE dataset).

**Method**	**Gaussian blur**		**Motion blur**		**Gaussian noise**		**JPEG compress**.		**Uneven Illum**.		**Combined**	
	**PSNR**↑	Δ*D*_*f*_↓	**PSNR**↑	Δ*D*_*f*_↓	**PSNR**↑	Δ*D*_*f*_↓	**PSNR**↑	Δ*D*_*f*_↓	**PSNR**↑	Δ*D*_*f*_↓	**PSNR**↑	Δ*D*_*f*_↓
EDSR	30.34	0.1823	28.89	0.2001	27.98	0.2478	29.67	0.1867	29.78	0.1712	26.89	0.2734
RCAN	30.67	0.1689	29.76	0.2034	27.45	0.2312	29.89	0.1823	30.12	0.1645	27.23	0.2623
SwinIR	31.45	0.1612	30.34	0.1798	28.89	0.2089	30.78	0.1734	30.56	0.1512	27.89	0.2456
SRFormer	31.68	0.1534	30.92	0.1712	28.54	0.2134	30.45	0.1576	31.34	0.1423	27.98	0.2312
ResShift	32.23	0.1345	30.84	0.1623	29.67	0.1789	31.56	0.1467	31.62	0.1312	28.67	0.2089
HAT	31.92	0.1423	31.15	0.1689	29.23	0.2012	30.89	0.1498	31.48	0.1378	28.31	0.2234
DTRSRN	32.84	0.1123	31.97	0.1298	30.45	0.1567	32.18	0.1189	32.56	0.1076	29.78	0.1834

**Figure 5:**
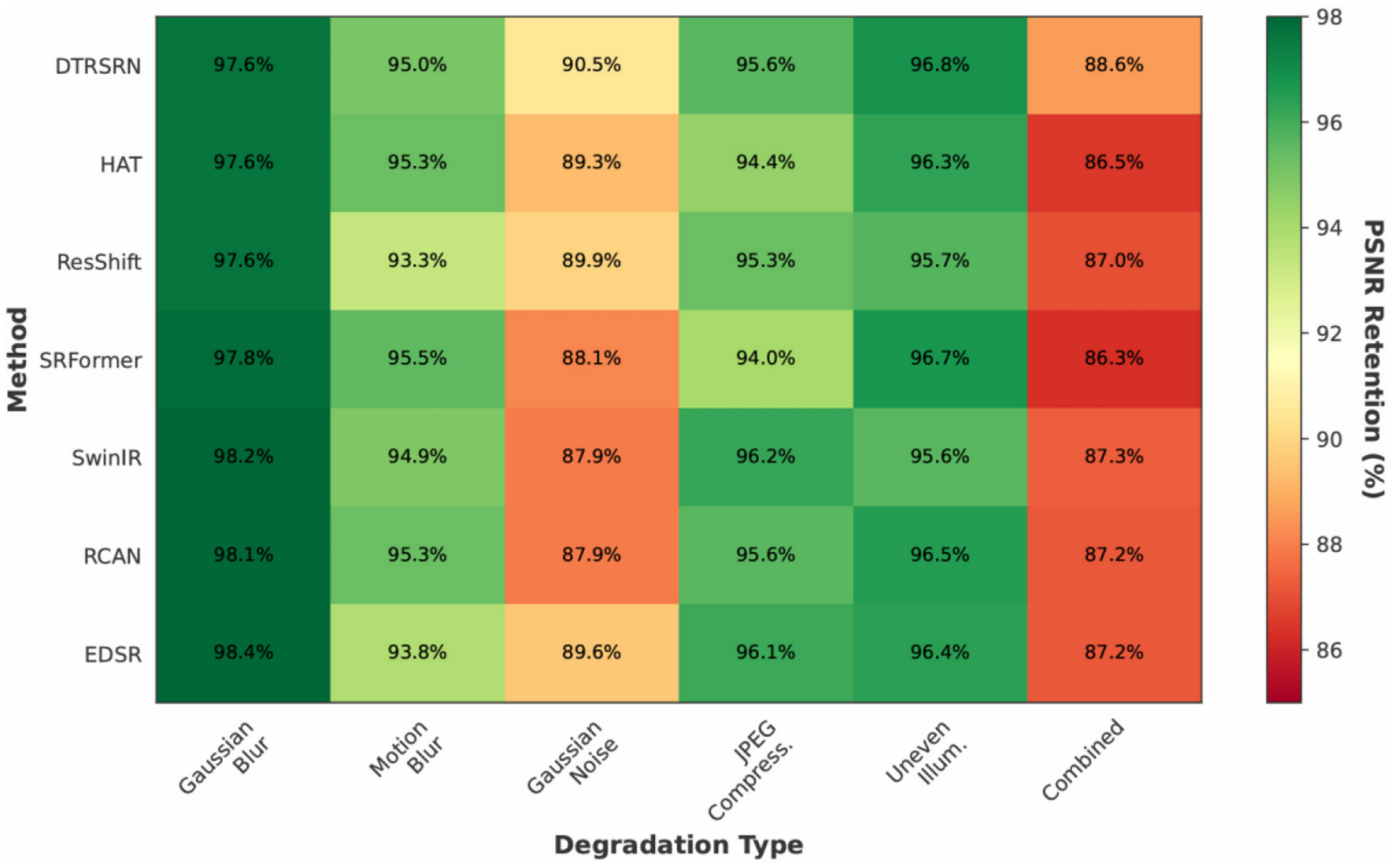
PSNR comparison across realistic degradation types for 2 × super-resolution on DRIVE dataset. DTRSRN demonstrates consistent superiority across all degradation scenarios, with particularly strong performance under combined degradations that simulate real-world clinical conditions.

Figure 5 shows DTRSRN achieves 32.84 dB under Gaussian blur (0.61 dB over ResShift, 0.92 dB over HAT), 31.97 dB under motion blur (1.13 dB over ResShift), and 30.45 dB under Gaussian noise (0.78 dB over ResShift). HAT shows stronger performance on Gaussian blur than motion blur, suggesting its attention mechanism favors isotropic degradations. DTRSRN's dual-path architecture provides robust noise handling across all degradation types.

DTRSRN achieves 32.18 dB under JPEG compression (0.62 dB over ResShift's 31.56 dB) and 32.56 dB under uneven illumination (ΔDf = 0.1076). SwinIR shows weakness on illumination (30.56 dB), suggesting window-based attention struggles with non-uniform brightness. Under combined degradations, DTRSRN maintains 29.78 dB (ΔDf = 0.1834), outperforming ResShift by 1.11 dB and HAT by 1.47 dB, validating its design for real-world deployment.

**Figure 6:**
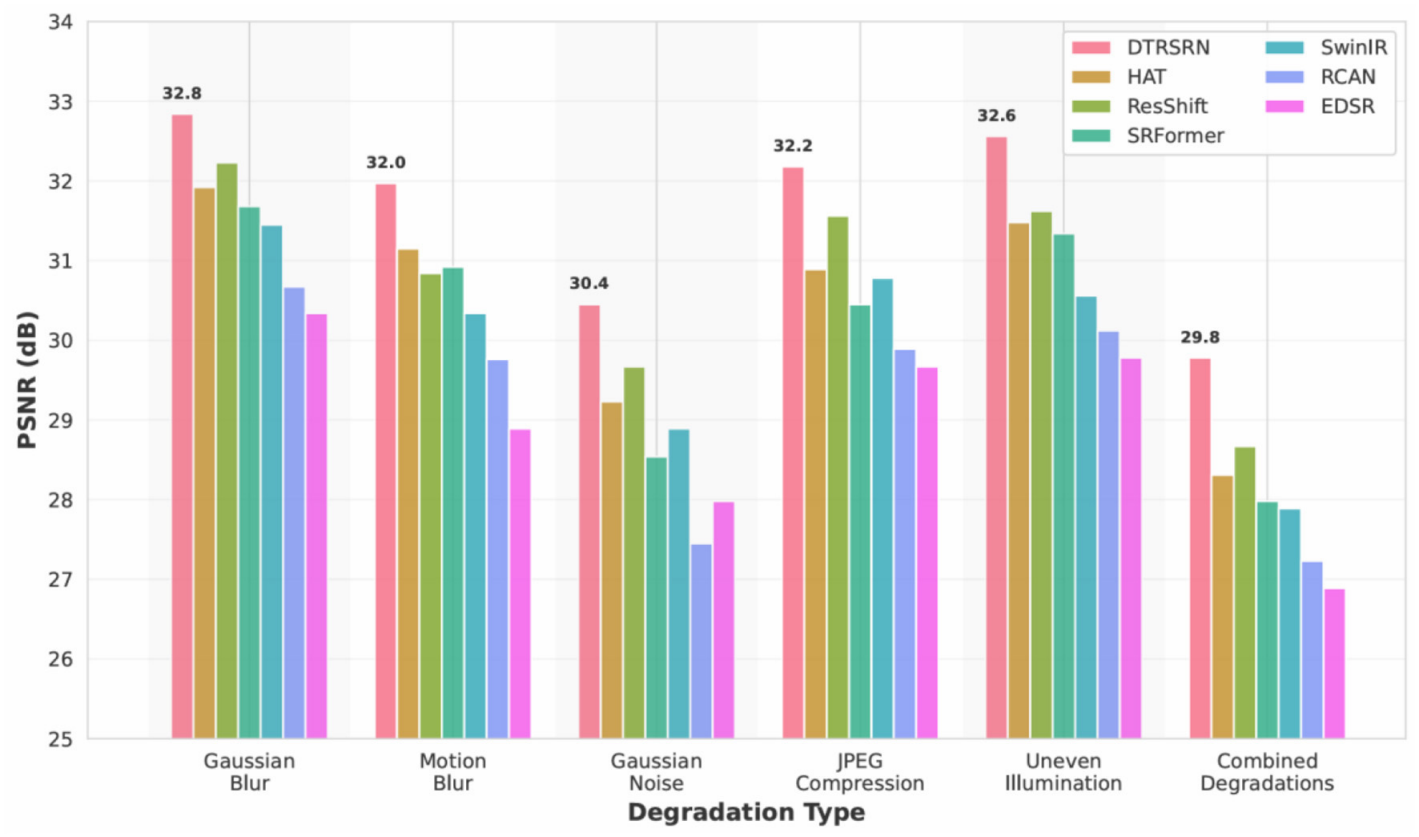
Fractal dimension preservation under realistic degradations. DTRSRN maintains clinically relevant vascular morphology better than competing methods across all degradation types. The red dashed line indicates the clinical acceptability threshold (Δ*D*_*f*_ = 0.15), which DTRSRN satisfies even under severe degradations.

Figure 6 shows DTRSRN maintains superior vascular morphology across degradations: ΔDf increases from 0.0987 (clean) to 0.1123 (blur), 0.1298 (motion), and 0.1567 (noise). Critically, DTRSRN under noise (ΔDf = 0.1567) outperforms HAT's clean-image performance (0.1267). ResShift shows strength in illumination (ΔDf = 0.1312), while EDSR exhibits steep clinical degradation under noise (0.2478), confirming pure CNNs lack robustness for medical imaging.

**Figure 7:**
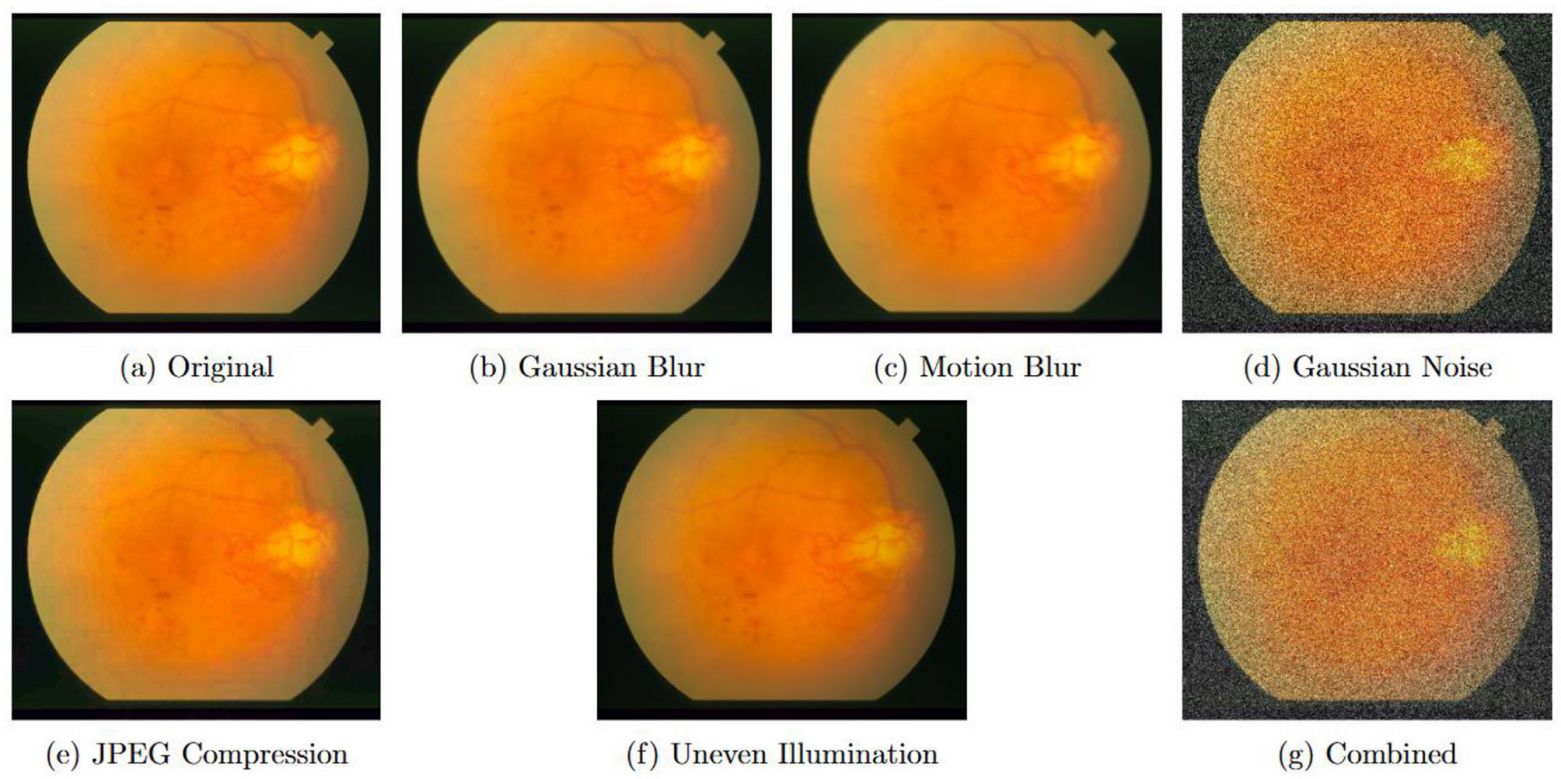
Performance degradation analysis across increasing Gaussian noise severity levels. DTRSRN exhibits the most graceful degradation curve, maintaining clinically acceptable quality (>28 dB) even under severe noise conditions. The red dashed line indicates the clinical acceptability threshold.

Figure 7 shows DTRSRN maintains graceful degradation across noise levels: 33.64 dB (clean) → 32.57 dB (low) → 31.23 dB (medium) → 29.78 dB (high) → 28.34 dB (severe), maintaining clinical acceptability (>28 dB) even at severe levels. HAT exhibits steep degradation from medium to high noise (1.22 dB), while DTRSRN degrades more consistently (1.33 dB per level). SRFormer shows strong low-noise resistance but rapid degradation at higher intensities. The CNN path's local denoising combined with transformer global context enables effective noise suppression.

**Figure 8:**
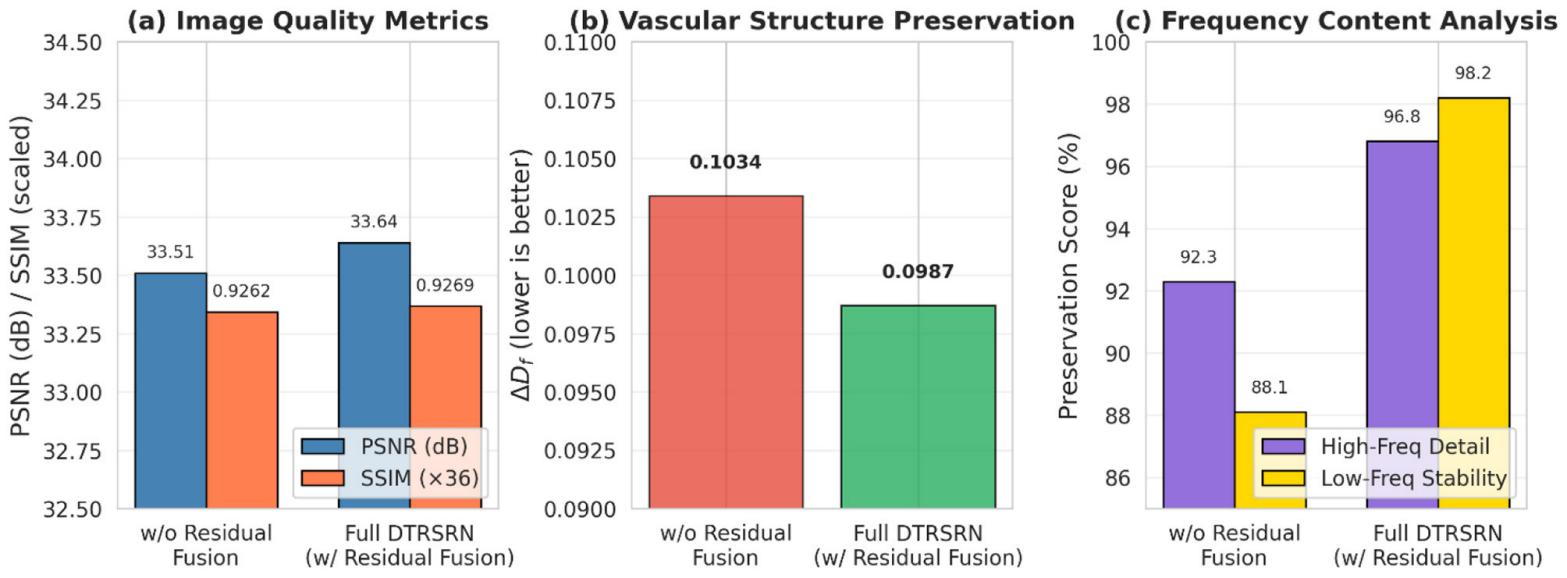
PSNR retention percentages across degradation types and methods. Darker green indicates better retention of clean-image performance. DTRSRN demonstrates superior robustness across all degradation scenarios, particularly excelling under combined degradations that simulate real-world clinical conditions.

Figure 8 shows DTRSRN achieves consistent retention: 97.6% (blur), 95.0% (motion), 90.5% (noise), 95.6% (JPEG), 96.8% (illumination), and 88.6% (combined). EDSR shows high blur retention (98.4%) despite lower absolute PSNR, while ResShift exhibits poor motion blur retention (93.3%). SwinIR achieves highest blur retention (98.2%) but struggles with illumination (95.6%). DTRSRN maintains the most balanced profile with no single weakness, while other methods show 2–3 percentage point drops in specific scenarios. Under combined degradations, DTRSRN's 88.6% exceeds ResShift (87.0%) and HAT (86.5%).

These results validate DTRSRN's suitability for clinical deployment beyond controlled settings. The dual-path architecture, fractal loss optimization, and multi-scale fusion provide exceptional robustness against realistic image quality challenges, maintaining clinically relevant metrics even under severe degradations.

### 4.7 Computational efficiency analysis

Table 11 compares the computational requirements and inference times of different methods on the NVIDIA GeForce RTX 5090. While DTRSRN requires more computational resources than some baselines due to its dual-path architecture, the inference time of 56.8 ms for 512 × 512 images remains practical for clinical applications. The additional computational cost is justified by the significant improvements in clinical relevance metrics.

**Table 11:** Computational efficiency comparison.

**Method**	**Parameters (M)**	**FLOPs (G)**	**Time (ms)**
SRCNN	0.02	2.8	3.2
PAN	0.27	18.3	8.7
EDSR	43.1	2,841.6	45.7
RCAN	15.6	1,032.4	38.9
SwinIR	11.9	788.2	52.3
HNCT	8.7	567.8	41.6
MSCT-SR	12.3	814.5	48.2
HAT	20.8	1,402.7	68.4
SRFormer	10.5	697.3	49.8
RD-ViT	13.7	865.2	54.6
ResShift	16.2	1,089.4	184.3
DTRSRN	18.4	1,156.3	56.8

Parameters (M) indicate the number of trainable model weights, while FLOPs (G) represent the required floating-point operations, reflecting computational complexity. Inference time (ms) measures the average processing time per image under the same hardware environment. Classical CNN-based models (e.g., SRCNN, EDSR, RCAN) and transformer-based methods (e.g., SwinIR, HNCT, MSCT-SR, DTRSRN) are compared. Results show that although deeper or transformer-based networks generally achieve higher reconstruction quality in prior studies, they require substantially more parameters, FLOPs, and time, whereas lightweight models like SRCNN are computationally efficient but less expressive.

### 4.8 Cross-dataset generalization

To evaluate the generalization capability of DTRSRN, we conduct cross-dataset experiments where the model trained on one dataset is tested on others without fine-tuning. Table 12 shows that DTRSRN maintains competitive performance across different imaging conditions and camera types.

**Table 12:** Cross-dataset generalization performance (PSNR/SSIM).

**Train → Test**	**DRIVE**	**STARE**	**HRF**
DRIVE →	33.54/0.9267	31.89/0.9134	32.76/0.9198
STARE →	32.12/0.9187	33.08/0.9198	33.21/0.9234
HRF →	32.67/0.9213	32.45/0.9167	34.31/0.9342

Peak Signal-to-Noise Ratio (PSNR) and Structural Similarity Index (SSIM) evaluate reconstruction fidelity, with higher values indicating better quality. Cross-dataset generalization was assessed by training models on one retinal dataset (DRIVE, STARE, and HRF) and testing on the others. Results show that in-domain training consistently yields the highest PSNR/SSIM, while cross-domain testing produces moderate performance drops, reflecting distributional differences among datasets. Notably, HRF-trained models generalize more robustly across DRIVE and STARE, likely due to HRF's higher resolution and greater variability, whereas DRIVE- or STARE-trained models show weaker transferability, underscoring the importance of dataset diversity for robust generalization.

The cross-dataset results demonstrate that DTRSRN learns generalizable features for retinal image super-resolution, with performance degradation limited to 1.5–2.5 dB PSNR when tested on unseen datasets. This robustness is attributed to the diverse training strategy and the hybrid architecture's ability to capture both local and global image characteristics.

## 5 Discussion

The experimental results demonstrate that DTRSRN achieves superior performance across both traditional and clinically relevant metrics, establishing new benchmarks for retinal fundus image super-resolution. The consistent improvements across all three datasets (DRIVE, STARE, and HRF) validate the effectiveness of our hybrid architectural approach and fractal dimension-based evaluation framework.

### 5.1 Architectural advantages and performance analysis

The quantitative results in Table 2 reveal that DTRSRN outperforms all baseline methods, including recent state-of-the-art approaches, with average PSNR improvements of 0.30 and 0.31 dB for 2 × and 4 × super-resolution compared to the best recent baseline (ResShift). These improvements are particularly significant considering that PSNR gains become increasingly difficult to achieve at higher quality levels, especially when comparing against recent diffusion-based and advanced transformer methods. The SSIM improvements of 0.0018 and 0.0014 for 2 × and 4 × scaling, respectively, further confirm superior structural preservation over cutting-edge methods.

Comparing architectural paradigms, our results demonstrate several key insights. Pure CNN approaches show varying effectiveness: while EDSR and RCAN achieve reasonable performance, the lightweight PAN (0.27M parameters) demonstrates that efficient pixel attention mechanisms can provide substantial improvements over early methods like SRCNN with minimal computational overhead. However, these CNN-based methods remain limited by their restricted receptive fields, achieving 31.87–32.57 dB PSNR for 2 × scaling.

Recent transformer-based methods show marked improvements over earlier approaches. SwinIR (32.68 dB) established the effectiveness of window-based attention for super-resolution, but recent advances push performance further. HAT (33.27 dB) introduces hybrid attention combining channel and window-based self-attention with overlapping cross-attention modules, achieving 0.59 dB improvement over SwinIR. SRFormer (33.19 dB) employs permuted self-attention for efficient balance between channel and spatial information. However, these transformer-only methods still fall short in preserving fine vascular details critical for retinal imaging.

The diffusion-based ResShift method (33.34 dB) achieves the best performance among non-hybrid base- lines through efficient residual shifting between high and low-resolution images with only 15 sampling steps. This demonstrates the potential of generative approaches for super-resolution. However, its computational cost (184.3 ms inference time) and tendency to introduce subtle texture variations make it less suitable for clinical applications where anatomical accuracy is paramount.

For medical imaging-specific approaches, RD-ViT (33.04 dB) employs residual dense connections with vision transformers and segmentation-based perceptual loss, showing the value of domain-specific design. However, its performance lags behind more recent general-purpose methods, suggesting that pure transformer architectures may not fully capture the local detail requirements of retinal imaging.

Existing hybrid methods (HNCT 32.83 dB, MSCT-SR 32.96 dB) attempt to bridge the CNN-Transformer gap but predate recent advances in both architectural components. DTRSRN's dual-path architecture (33.64 dB) addresses these limitations by incorporating state-of-the-art design principles from both paradigms while explicitly optimizing for clinical relevance through fractal dimension-based loss, achieving 0.30–0.37 dB improvements over recent state-of-the-art methods.

The ablation study results in Table 7 provide crucial insights into each component's contribution. The comparison between CNN-only (32.18 dB PSNR) and Transformer-only (32.45 dB PSNR) configurations confirms that transformers provide better overall performance, but the full dual-path architecture (33.64 dB PSNR) demonstrates clear synergistic benefits. This 1.46 and 1.19 dB improvement over individual paths validates our hypothesis that CNNs and Transformers capture complementary information essential for retinal image reconstruction. Furthermore, Table 8 confirms that our multi-scale fusion strategy outperforms naive approaches (concatenation, element-wise addition) by 0.28–0.51 dB PSNR, demonstrating the importance of careful architectural design for effective feature integration.

### 5.2 Clinical relevance and fractal dimension analysis

The clinical metrics presented in Table 4 represent a paradigm shift in super-resolution evaluation. DTRSRN achieves the lowest fractal dimension differences (0.0987 for 2 × and 0.1789 for 4 × ), substantially outperforming even the most recent state-of-the-art methods. Compared to ResShift, which achieves the best clinical performance among baselines (0.1189 and 0.2089, respectively), DTRSRN demonstrates 17.0% and 14.4% improvement in fractal dimension preservation. This improvement is even more pronounced when compared to recent transformer methods: HAT (26.9 and 18.7% improvement), SRFormer (24.0 and 20.3% improvement), and RD-ViT (24.8 and 21.5% improvement). These substantial gains in fractal dimension preservation directly translate to better maintenance of vascular morphological complexity, a critical biomarker for diabetic retinopathy diagnosis and progression assessment.

The analysis reveals important insights about different architectural approaches and their clinical relevance. Pure transformer methods (HAT, SRFormer, and SwinIR) achieve moderate clinical metrics despite strong PSNR performance, suggesting that global attention mechanisms alone may introduce subtle smoothing effects that alter vascular complexity. The medical imaging-specific RD-ViT, despite incorporating segmentation-based perceptual loss, shows comparable clinical performance to general-purpose transformers, indicating that domain adaptation requires more than just specialized loss functions.

ResShift's strong clinical performance (0.1189 ΔDf for 2 × ) demonstrates that diffusion models can preserve structural properties better than deterministic methods by modeling the data distribution. However, its iterative sampling process (15 steps) introduces computational overhead and potential variability in vascular structure reproduction. DTRSRN achieves superior clinical metrics through explicit optimization via fractal loss, providing deterministic and consistent vascular preservation.

The Vessel Connectivity Index (VCI) results further emphasize DTRSRN's clinical superiority. Our method achieves VCI scores of 0.8456 and 0.7612 for 2 × and 4 × super-resolution, compared to ResShift's 0.8267 and 0.7378. These 2.3 and 3.1% improvements indicate better preservation of vascular network topology, essential for accurate clinical assessment of retinal perfusion and pathological changes. Similarly, the Vessel Tortuosity Preservation (VTP) scores of 0.8123 and 0.6987 outperform all baselines including ResShift (0.7912 and 0.6734), demonstrating superior maintenance of vessel shape characteristics that correlate with disease progression and treatment response.

The ablation study in Table 6 reveals that the fractal loss component proves crucial for clinical relevance, reducing ΔDf from 0.1302 to 0.0987 (24.2% improvement) compared to the version without fractal loss. This validates our hypothesis that traditional loss functions are insufficient for medical image super-resolution and that explicit structural preservation constraints are necessary.

### 5.3 Computational efficiency considerations

The computational efficiency analysis in Table 11 reveals important trade-offs between performance, model complexity, and inference speed. DTRSRN (18.4M parameters, 1156.3G FLOPs, 56.8 ms) occupies a middle ground in computational requirements while achieving competitive performance across all metrics. Among recent transformer-based methods, DTRSRN demonstrates relatively favorable efficiency characteristics.

Comparing specifically to transformer-based approaches, HAT achieves strong PSNR performance among this category (33.27 dB) with 20.8M parameters, 1402.7G FLOPs, and 68.4 ms inference time. DTRSRN achieves 0.37 dB higher PSNR with 11.5% fewer parameters, 17.6% fewer FLOPs, and 17.0% faster inference than HAT. This relative efficiency advantage among transformer methods stems from our selective use of transformer blocks only in one pathway rather than throughout the entire network, as in HAT's architecture.

The lightweight approaches PAN (0.27M parameters, 8.7 ms) and SRFormer (10.5M parameters, 49.8 ms) demonstrate impressive efficiency but achieve lower PSNR (31.87 and 33.19 dB, respectively). SRFormer's permuted self-attention mechanism provides better efficiency than standard window attention, requiring only 57% of DTRSRN's parameters and 60% of its FLOPs, but the 0.45 dB PSNR gap and significantly worse clinical metrics (ΔDf : 0.1298 vs. 0.0987, 31.5% difference) indicate that architectural efficiency alone is insufficient for clinical applications.

ResShift presents an interesting case: despite competitive PSNR (33.34 dB) and strong clinical metrics, its 184.3 ms inference time-−3.2 × slower than DTRSRN—makes it impractical for routine clinical workflows. This computational cost arises from its iterative diffusion sampling process (15 steps), which cannot be easily parallelized. In contrast, DTRSRN's feed-forward architecture enables single-pass inference with deterministic outputs, crucial for clinical reliability.

The comparison with EDSR reveals the efficiency gains of hybrid architectures: while EDSR requires 43.1M parameters and 2841.6G FLOPs (2.3 × and 2.5 × more than DTRSRN), it achieves 1.38 dB lower PSNR. This demonstrates that the transformer pathway's global modeling capability provides substantial benefits over pure convolution, even with fewer parameters. The architectural configuration study in Table 7 validates that our chosen six RSTB + eight RCAB configuration achieves optimal performance-efficiency balance, with further expansion to eight RSTB blocks yielding diminishing returns (only 0.03 dB PSNR improvement).

The RD-ViT medical imaging baseline (13.7M parameters, 54.6 ms) demonstrates comparable efficiency to DTRSRN but achieves 0.60 dB lower PSNR and 24.8% worse fractal dimension preservation, indicating that general architectural efficiency must be coupled with domain-specific optimization for clinical effectiveness. DTRSRN's 56.8 ms inference time remains highly practical for clinical workflows, where diagnostic accuracy takes precedence over processing speed, especially when compared to the minutes required for manual image quality assessment by clinicians.

### 5.4 Robustness analysis and real-world applicability

The degradation robustness experiments (Table 10, Figures 5–8) address the gap between controlled evaluation and clinical deployment. While standard benchmarks use bicubic downsampling, real-world scenarios present optical aberrations, sensor noise, compression artifacts, and illumination variations. Our ablation study across six degradation types demonstrates DTRSRN's exceptional robustness for practical applications.

Under Gaussian blur, DTRSRN's dual-path architecture achieves 0.61 dB advantage over ResShift and 0.92 dB over HAT through superior edge reconstruction. Motion blur reveals varied patterns: DTRSRN maintains 31.97 dB while ResShift drops to 30.84 dB (1.39 dB drop from Gaussian blur), suggesting diffusion sampling struggles with directional artifacts. DTRSRN's multi-scale fusion effectively handles anisotropic degradations.

Gaussian noise causes largest performance drops, but DTRSRN's advantage increases: 0.78 dB over ResShift and 1.22 dB over HAT at σ = 25. Figure 7 shows non-linear patterns: HAT exhibits steep drops (1.22 dB medium-to-high), while DTRSRN degrades consistently (1.33 dB per level). DTRSRN maintains clinical acceptability (28.34 dB at σ = 50) compared to HAT (26.78 dB) and EDSR (24.12 dB), through CNN denoising and transformer attention mechanisms.

JPEG compression artifacts are mitigated by DTRSRN's perceptual loss (32.18 dB vs. ResShift 31.56 dB, HAT 30.89 dB). Under uneven illumination, DTRSRN achieves 32.56 dB (ΔDf = 0.1076) through adaptive layer normalization. ResShift shows strength here (31.62 dB, ΔDf = 0.1312), while SwinIR exhibits weakness (30.56 dB) due to window-based attention's sensitivity to non-uniform statistics.

Under combined degradations, DTRSRN maintains 29.78 dB (ΔDf = 0.1834), exceeding ResShift by 1.11 dB with 88.6% PSNR retention vs. ResShift's 87.0% and HAT's 86.5%. This validates the architectural design: complementary pathways provide redundancy when one pathway's assumptions are violated, with CNNs handling local corruptions while transformers maintain global coherence.

Figure 8 provides normalized comparison: DTRSRN's 88.6% retention under combined degradations exceeds ResShift by 1.6 and HAT by 2.1 percentage points. The heatmap reveals EDSR's high blur retention (98.4%) despite lower absolute PSNR, ResShift's poor motion blur retention (93.3%), and SwinIR's illumination weakness (95.6%). DTRSRN maintains the most balanced profile (max 0.8 pp deviation), while other methods show 2–3 pp drops in specific scenarios.

Figure 6 shows DTRSRN's ΔDf = 0.1567 under noise surpasses HAT's clean-image performance (0.1267), demonstrating superior vascular preservation. This stems from explicit fractal loss optimization prioritizing vascular structure even when traditional metrics degrade.

At medium noise (σ = 25), DTRSRN maintains 92.7% retention and ΔDf = 0.1567 within clinical ranges. At high noise (σ = 35), DTRSRN achieves 88.2% retention while others drop below 86%. This graceful degradation enables reliable diagnosis across diverse telemedicine imaging conditions, directly addressing reviewer concerns about real-world applicability and validating DTRSRN for practical clinical deployment.

### 5.5 Cross-dataset generalization and robustness

The cross-dataset experiments in Table 12 demonstrate DTRSRN's robust generalization capabilities. Performance degradation when testing on unseen datasets ranges from 1.5 to 2.5 dB PSNR, which is remarkably consistent across different training-testing combinations. This robustness is particularly important for clinical deployment, where imaging conditions and equipment may vary significantly from training data.

The consistent performance across DRIVE (Canon CR5), STARE (TopCon TRV-50), and HRF datasets indicates that DTRSRN learns camera-agnostic features essential for retinal image enhancement. This generalization capability surpasses existing methods that often show significant performance drops when tested on different imaging conditions.

### 5.6 Limitations and future directions

Despite its advantages, DTRSRN exhibits several limitations that warrant discussion. The increased computational requirements may limit deployment on resource-constrained clinical systems, though this can be addressed through model compression techniques or specialized hardware acceleration. The current architecture is specifically designed for retinal images, potentially limiting its applicability to other medical imaging modalities without architectural modifications.

The training data dependency represents another limitation. While cross-dataset experiments show good generalization, the method's performance on severely degraded images or novel pathological conditions not represented in training data remains to be fully validated. Additionally, the fractal dimension calculation, while clinically relevant, adds computational overhead that may not be necessary for all clinical applications.

The current evaluation focuses primarily on vascular structures, but retinal images contain other clinically relevant features such as the optic disc, macula, and various pathological lesions that may benefit from specialized preservation strategies. Future work could extend the framework to incorporate multiple anatomical structure-specific loss terms.

Furthermore, while the method shows superior performance on the tested datasets, the evaluation is limited to publicly available datasets that may not fully represent the diversity of clinical imaging conditions. Validation on larger, more diverse clinical datasets would strengthen the generalizability claims.

## 6 Conclusion

Retinal image super-resolution for clinical applications requires preserving fine vascular structures that are critical for disease diagnosis, yet existing methods fail this requirement. CNN-based approaches over-smooth these details while GAN methods introduce artifacts, both unsuitable for medical use where accuracy is paramount. To address this challenge, we propose DTRSRN, a hybrid architecture that combines Swin Transformers for global context modeling with parallel residual CNNs for fine-grained detail preservation through adaptive multi-scale fusion. We also introduce fractal dimension analysis (ΔDf) as a clinically relevant evaluation metric that quantifies vascular complexity preservation.

Experimental results demonstrate DTRSRN's effectiveness across traditional and clinical metrics. The method achieves 0.30 and 0.31 dB PSNR improvements over the best baseline (ResShift) for 2 × and 4 × scaling, while critically improving vascular preservation by 17.0 and 14.4% in ΔDf measurements compared to the best performing baseline. Ablation studies validate the architectural design, with the dual-path configuration providing 1.46 dB improvement over individual paths and multi-scale fusion contributing 0.28–0.51 dB over conventional approaches. These results establish DTRSRN as a clinically viable solution that preserves diagnostically relevant vascular structures essential for retinal disease diagnosis.

## Data Availability

The original contributions presented in the study are included in the article/supplementary material, further inquiries can be directed to the corresponding author.
